# An Integrative multi-lineage model of variation in leukopoiesis and acute myelogenous leukemia

**DOI:** 10.1186/s12918-017-0469-2

**Published:** 2017-08-25

**Authors:** Joyatee M. Sarker, Serena M. Pearce, Robert P. Nelson, Tamara L. Kinzer-Ursem, David M. Umulis, Ann E. Rundell

**Affiliations:** 1 Weldon School of Biomedical Engineering, Purdue University, 206 S. Martin Jischke Drive, West Lafayette, 47906 IN USA; 2 Department of Medicine and Pediatrics, Divisions of Hematology/Oncology, Indiana University School of Medicine, 535 Barnhill Dr., Ste. 473, Indianapolis, 46202 IN USA; 3 Ag. and Biological Engineering, Purdue University, 206 S. Martin Jischke Drive, West Lafayette, 47906 IN USA

**Keywords:** Acute myelogenous leukemia, Mathematical model, Personalized medicine, Hematopoiesis, Leukopoiesis

## Abstract

**Background:**

Acute myelogenous leukemia (AML) progresses uniquely in each patient. However, patients are typically treated with the same types of chemotherapy, despite biological differences that lead to differential responses to treatment.

**Results:**

Here we present a multi-lineage multi-compartment model of the hematopoietic system that captures patient-to-patient variation in both the concentration and rates of change of hematopoietic cell populations. By constraining the model against clinical hematopoietic cell recovery data derived from patients who have received induction chemotherapy, we identified trends for parameters that must be met by the model; for example, the mitosis rates and the probability of self-renewal of progenitor cells are inversely related. Within the data-consistent models, we found 22,796 parameter sets that meet chemotherapy response criteria. Simulations of these parameter sets display diverse dynamics in the cell populations. To identify large trends in these model outputs, we clustered the simulated cell population dynamics using k-means clustering and identified thirteen ‘representative patient’ dynamics. In each of these patient clusters, we simulated AML and found that clusters with the greatest mitotic capacity experience clinical cancer outcomes more likely to lead to shorter survival times. Conversely, other parameters, including lower death rates or mobilization rates, did not correlate with survival times.

**Conclusions:**

Using the multi-lineage model of hematopoiesis, we have identified several key features that determine leukocyte homeostasis, including self-renewal probabilities and mitosis rates, but not mobilization rates. Other influential parameters that regulate AML model behavior are responses to cytokines/growth factors produced in peripheral blood that target the probability of self-renewal of neutrophil progenitors. Finally, our model predicts that the mitosis rate of cancer is the most predictive parameter for survival time, followed closely by parameters that affect the self-renewal of cancer stem cells; most current therapies target mitosis rate, but based on our results, we propose that additional therapeutic targeting of self-renewal of cancer stem cells will lead to even higher survival rates.

**Electronic supplementary material:**

The online version of this article (doi:10.1186/s12918-017-0469-2) contains supplementary material, which is available to authorized users.

## Background

Acute myelogenous leukemia (AML) is a cancer of the white blood cells stemming from the myeloid lineage that produces cells including neutrophils and monocytes [1]. On average, four in 100,000 individuals will develop AML, with a median age of 67 years at diagnosis. AML has the highest mortality rate of all of the different types of leukemia [2]. The French-American-British (FAB) co-operative group identified eight different subcategories of AML, M0-M7, based on the specific cell population from which AML arises [3]. More recent classification systems now exist that include other criteria besides white cell morphology [4, 5]. Regardless, the majority of patients with AML receive essentially identical induction chemotherapy with drugs that target DNA replication [6]. Unfortunately, for the patients over 55 years of age diagnosed with AML, the 5-year survival rate is 10% and the relapse rate is 80% [7]. Given the variability of AML progression among patients, understanding determinants of disease progression will lead to therapeutic advances that result in lower rates of relapse and higher rates of survival.

Computational modeling is an effective tool to personalize therapies for improved patient outcomes. Model-based personalized therapy has improved several medical interventions including: glucose control in type I diabetes [8, 9]; automatic control of anesthesia dosage [10–12]; and pacemaker control of heart rate variability [13]. Presently, AML treatment is not benefiting from model-based personalized approaches, in part due to a lack of multi-lineage AML computational models [14, 15]. Key features of a computational model of hematopoiesis includes: normal formation of mature cells from hematopoietic stem cells (HSCs) [16–24]; abnormal development leading to leukemia [18, 25–27]; treatment using chemotherapy or transplantation [17, 25, 26]; movement of cells between the bone marrow/tissue and the peripheral blood where AML is frequently measured [20, 28–35]; and the feedback signals from cytokines that participate in the regulation of hematopoiesis [20, 25, 26, 28]. Previous models that contain various elements of these key features exist (summarized in Additional file 1: Table S1 in Supplement 1). Here, we focus on the development of a multi-lineage AML model that captures patient variability and use the model to identify patient sub-types and identify potential novel therapeutic targets.

In this work, we develop a semi-mechanistic multi-lineage multi-compartment computational model of hematopoiesis and AML. The model describes the interactions of various lineages of hematopoietic stem cells, including neutrophils, lymphocytes, and monocytes. Patient data is used to constrain the model, creating a large number of data-consistent solutions that differ in their responses to chemotherapy. We apply the constraints from patient data to characterize normal hematopoiesis versus leukemic differentiation and growth in unique parameter sets representing 22,796 simulated patient dynamics. To identify trends in dynamical variability, we clustered the simulated dynamics into thirteen ‘representative patient’ groups. We find that our mathematical model can be used to demonstrate the typical variation in the dynamics of AML progression and patient clinical outcomes. Using sensitivity analysis and correlation analysis, we identified important parameters whose variation most affects the development of leukemia.

## Methods

**Fig. 1:**
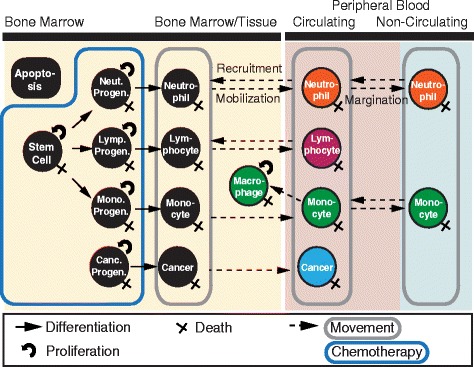
Hematopoiesis model overview. Each state of the model is represented with a circle. Mature cells are colored and immature cells are shown with black backgrounds. The compartments of the model are labeled on the top of the figure and indicated with solid colored rectangles. The peripheral blood is divided into two compartments: circulating blood and non-circulating blood, or the marginal pool. Solid arrows represent the differentiation from one state to the state to which the arrow points. A curved arrow indicates self-renewal capability. The group of cells that move are boxed by a gray rounded rectangle. An ‘x’ on the bottom right of a state node represents natural death. The cells that are affected by chemotherapy are encapsulated by the blue rounded rectangular shape. Cells that are undergoing apoptosis and will be cleared by macrophages are in the ‘Apoptosis’ compartment

Herein, we develop a semi-mechanistic multi-lineage model of leukopoiesis and the formation of AML that includes the following: (1) variability in healthy and malignant white blood cell maturation; (2) interactions between progenitor cells and differentiated cells; (3) interactions amongst macrophages, neutrophils, and lymphocytes; and (4) dynamics of hematopoiesis and AML progression (Fig. 1; model equations in Additional file 1: Supplement 2). This ordinary differential equations (ODE) model of hematopoiesis describes the physiological processes of cell maturation, cell mobility between different blood compartments in the body, interactive responses to cytokines and the cells that produce them, cellular activation and suppression, and cell loss due to natural death or chemotherapy.

### Model Configuration

The leukopoiesis process consists of a set of discrete stages of cellular differentiation. There are three different stages of cell development representing the maturation of the stem cells into mature leukocytes (Fig. 1, solid black circles). The first stage contains the hematopoietic stem cells (HSCs) that either asymmetrically self-renew or differentiate. We assume this HSC compartment consists of many stages of cells that have the capability to differentiate into various lineages, as previous work has shown that the dynamics of mature cells are appropriately represented in a two-compartment system [36–38]. The stem cells proportionally differentiate into distinct progenitors, including common lymphoid progenitors and granulocyte/myeloid progenitors that then clonally amplify their branching lineages [39]. All three lineages are necessary to demonstrate lineage dominance and cell-cell interactions amongst the three most-commonly measured white blood cell types (see Additional file 1: Supplement 3, Table S5 for further justification of these three lineages). Other multi-lineage models do not consider these three lineages [21–24]. Granulocyte/myeloid progenitors produce mature neutrophils and monocytes, which have relatively short life-spans and are replaced daily. Lymphoid progenitors branch into immature B-lymphocytes and T-lymphocytes, yielding more complex and less predictable dynamics than those of myeloid cells. Thus, we focused on studying myeloid cell production, destruction, and deviations associated with myeloid leukemias instead of the less predictable lymphoid leukemias. The process of proliferation and differentiation in our model ensures a physiological mechanism that repopulates the bone marrow after chemotherapy and ensures that the formation of a single mutated cell in the HSC compartment can result in AML.

Mature cells are maintained in three different compartments: the bone marrow or tissue (yellow compartment in Fig. 1), the circulating peripheral blood (red compartment in Fig. 1), and the non-circulating peripheral blood, or marginal pool (blue compartment in Fig. 1). Cells migrate from both the bone marrow and marginal pool to the peripheral blood and are recruited to the bone marrow or marginal pools from the peripheral blood [40–42]. Since the concentration of normal white blood cells in the bone marrow and peripheral blood may vary over several orders of magnitude [40], it is essential to compute the dynamics of the proliferation of cells within the bone marrow and the mobilization of cells. Other multi-lineage models do not demonstrate movement of cells amongst compartments [21–24], and mobilization is typically limited to neutrophils or monocytes [20, 27–31, 33–35]. Additionally, neutrophils and monocytes also remain in non-circulating peripheral blood pools, or marginal pools, to allow for massive demargination upon a trigger event, such as chemotherapy [40, 43].

Our final model contains 17 states that represent cell populations and 51 parameters. The equations for this model can be found in Additional file 1: Supplement 2, and the model is depicted in Fig. 1. Details of these biophysical processes that regulate cellular dynamics are in Additional file 1: Supplement 3. The parameter values can be found in Additional file 1: Supplement 4. The general form of the model for each cellular state is a sum of all of the production terms, the movement terms, and the loss terms (Eq. 1).

1 $$ \begin{aligned} \frac{dX_{0}}{dt} &= +p_{0}*X_{0} +s_{-1}*X_{-1} - s_{0}*X_{0} + m_{-1}*X_{bm/pb}\\ &\quad- r_{0}*X_{0} - d_{0}*X_{0} \end{aligned}   $$

Here, *X*_0_ is the state of interest; *p*_0_ is the proliferation term, typically described as asymmetric self-renewal (circular arrows in Fig. 1; based on [19]; described further in Additional file 1: Supplement 3); *s*_−1_ is the differentiation (specialization) term from a stem cell or progenitor state, *X*_−1_, to the state of interest, *X*_0_; *s*_0_ is the differentiation term from the state of interest, *X*_0_, to a more mature state (straight solid arrows in Fig. 1); *m*_−1_ is the mobilization or margination term from the corresponding bone marrow or peripheral blood state, *X*_*bm*/*pb*_, to the state of interest, *X*_0_ (forward dashed arrows in Fig. 1); *r*_0_ is the recruitment term of *X*_0_ to another compartment (backward dashed arrows in Fig. 1); and *d*_0_ is the loss, or death rate (x’s in Fig. 1). Further details of this model are described in Additional file 1: Supplement 3. A few key physiological details, including feedback and cell-cell interactions in the multi-lineage model, are described below.

### Feedback

Cells secrete complex cytokines and chemokines that play a pivotal role in hematopoiesis. Cytokines regulate different processes that occur in the hematopoietic system, such as self-renewal and movement [44]. Mutations in the production or signaling of cytokines can lead to negative outcomes, such as the formation of AML [45–48]. These cytokines are not specified in our model, but are assumed to be proportionate to the number of cytokine releasing cells (i.e. macrophages). This is incorporated into our model using Michaelis-Menten type kinetics to modify the rates of cytokine-targeted processes. The general form of negative feedback is in Eq. 2 and the general form of positive feedback is in Eq. 3.

2 $$  \frac{k}{k + C}rX  $$

3 $$  \frac{C}{k + C}rX  $$

Here, *k* is the Michaelis-Menten constant; *C* is the concentration of the cell state that produces cytokines for feedback; *r* is the rate the feedback modifies; and *X* is the cell state targeted by cytokines. To model the formation of AML from monocytes, we simulated the multiple-hit hypothesis and assumed all feedback signals did not effect a single normal monocyte progenitor, as expected from literature [49].

Inhibitory feedback prevents cells from growing uncontrollably in any state. Thus, our model has several inhibition mechanisms to regulate the concentration of cells. In particular, inhibition feedback modifies every rate except cell death in our model. All movement is inhibited by the concentration of cells in the compartment to which cells are moving to prevent overcrowding using Michaelis-Menten type kinetics (Eq. 2). Proliferation and differentiation are hindered by inhibiting the associated self-renewal probability of stem cells [19]. For progenitor cells, this inhibition occurs from cytokines produced by mature cells of the same lineage in the peripheral blood [50]. However, for stem cell proliferation and differentiation, this inhibition occurs from a scaled combination of the concentration of stem cells to ensure that sufficient stem cells are in the hematopoietic system and the concentration of cells in the bone marrow (yellow compartment in Fig. 1) do not exceed the capacity of the marrow. All negative feedback signals are pictorially described in Additional file 1: Figure S1. The derivation of both the form for asymmetric self-renewal and the capacity of the bone marrow are in Additional file 1: Supplement 3.

In contrast, several physiological processes in hematopoiesis are triggered by positive feedback. In response to an inflammatory event, mature white blood cells proliferate to accommodate this response. We include two states in our model that demonstrate the effect of debris clearance due to chemotherapy. Monocytes are recruited into the bone marrow to become activated macrophages to help clear excessive cellular debris [41], which we have modeled as the ‘Apoptosis’ state. Macrophages assist in recruiting other white blood cells, such as lymphocytes, neutrophils, and monocytes, during inflammation or other diseases [41], which we have also incorporated into the model. Positive feedback is used in our model to demonstrate several processes, including inducing proliferation of macrophages by apoptotic debris; promoting the recruitment of neutrophils, lymphocytes, and other monocytes into the tissue in response to high levels of macrophages; and increasing the clearing rate of apoptosis due to a high level of macrophages. All positive feedback signals are pictorially described in Additional file 1: Figure S2. Additional file 1: Table S4 in Supplement 2 describes each of the feedback processes in detail.

## Results

### Parameter selection

**Fig. 2:**
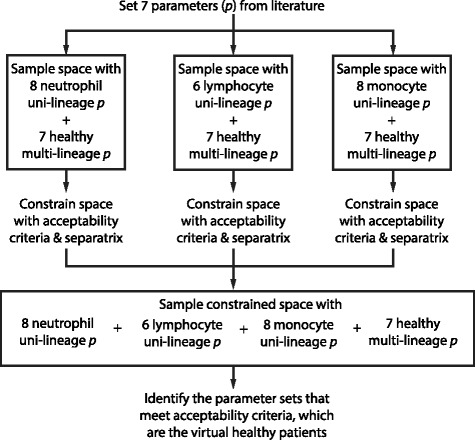
Identifying representative patients using qualitative and quantitative model features. Flowchart of the description of constraining uni-lineage and multi-lineage parameter spaces. After setting parameters from literature, parameters specific to each uni-lineage are sampled along with the parameters that are specific to stem cells. We characterize the simulations from these sampled parameter spaces as acceptable or unacceptable from the dynamic acceptability criteria in Table 1 and constrain the parameter space to reflect acceptable solutions. We re-sampled the constrained parameter space of all three lineages and simulated with the multi-lineage model. The simulations that met the acceptability criteria were used for further analysis

The model has 51 parameters and 17 states that describe the cell concentrations of various white blood cells and leukemia (Fig. 1) and reflects seven qualitative and quantitative features found in patient responses to chemotherapy from literature (Table 1). Seven of these parameters were found from literature: the death rates of progenitor cells [51], mature neutrophils [28, 52, 53], mature lymphocytes in the bone marrow [54] and peripheral blood [25, 55], mature monocytes in the bone marrow [28, 56, 57] and peripheral blood [56], and macrophages [58]. We fit the remaining 44 uncertain parameters to reflect the seven dynamic acceptability criteria (Table 1) of our model; these parameter values are in Additional file 1: Supplement 4. Parameter optimization proceeded in sequence by first identifying data-consistent parameters in the uni-lineage models and then extending the identified parameters to the integrated multi-lineage model (Fig. 2).

**Table 1:** Capabilities of multi-lineage hematopoiesis model

Healthy dynamical acceptability criteria
1. Peripheral blood recovers > 80 cells/ *μ*L [58]
2. Stem cells recover > 1 cell/ *μ*L [58]
3. Final dynamic values are within acceptable ranges (Additional file 1: Supplement 4) [40, 59]
4. Marginal pool is within one order of magnitude of peripheral blood [43]
5. Cell counts reduce with chemotherapy to < 20% of original value [63]
6. Recovery overshoot < 12 times value five days after overshoot [hematopoietic stem cell transplant patient data from Dr. Robert Nelson, shown in Additional file 1: Supplement 5].
7. Amplitude of the second peak of oscillations deviates < 18% from the amplitude of the first peak [cutoff calculated, explained in Additional file 5: Supplement 5].

**Fig. 3:**
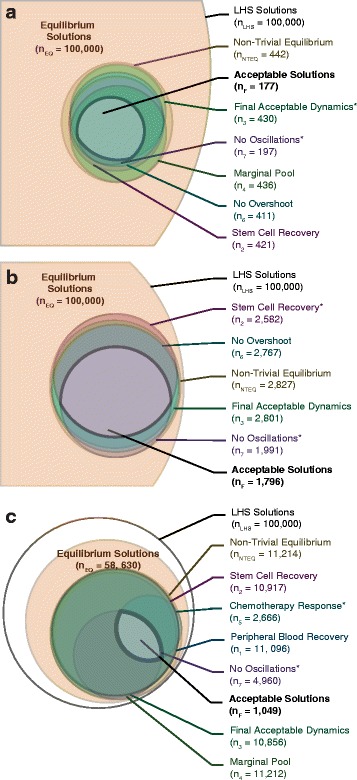
Parameter space constraints for uni-lineage models using dynamic acceptability criteria. Each circle represents the number of simulations that met the acceptability criteria indicated by the corresponding color-coded label. Overlapping circles demonstrate the region in which simulations met multiple criteria. Circles with straight edges represent an inset of the original circle and has been scaled to show other circles more clearly. **a** shows the acceptability for neutrophil uni-lineage solutions (*n* = 177 total acceptable), **b** shows the acceptability for the lymphocyte uni-lineage solutions (*n* = 1796 total acceptable), and **c** shows the acceptability for monocyte uni-lineage solutions (*n* = 1049 total acceptable). In **a** and **b** the parameter space constrained by the dynamical acceptability criteria are shown as an inset to distinguish amongst the constrained parameter sets. The two criteria that were salient in determining final total acceptability are indicated with asterisks. The subscripts on the ‘n’s correspond to the numbered criteria from Table 1

Qualitative features and properties and quantitative patient data were used to test models and optimize parameters. However, given the amount of patient-to-patient variability, this only informs regions of the parameter space consistent with observed clinical data as opposed to optimized parameter values. We explored for parameters in a nested approach, where uni-lineage models were evaluated independently before being combined with the multi-lineage model (Fig. 2). Specifically, we were interested in demonstrating a realistic range of patient white blood cell counts and the rates that dictate those counts. Manesso et al. also used a “divide et impera" step-wise approach to parameterizing their multi-lineage model to demonstrate variability in [23]; however, a key difference of their model to ours is that we searched for both initial conditions and parameter sets that met expected steady state configurations instead of using various parameter set perturbations on one initial condition. In order to find these initial conditions and parameter values, we searched over large parameter ranges for the 44 undetermined parameters using MATLAB’s lsqnonlin search algorithm. Self-renewal fractions were bound between 0.5 and 1 to maintain stem cell mass without becoming zero. Nominal values for the remainder of the parameters were approximated based on expected dynamics. We searched between 1/5 to 5 times these nominal values to determine the bounds of the parameter space. We simulated 100,000 Latin Hypercube samples, an efficient, semi-random but uniform method of sampling, in log space for each uni-lineage model (Fig. 3, *n*_*LHS*_). Almost all of the Latin Hypercube samples produced non-zero equilibrium solutions for further analysis (Fig. 3, *n*_*NTEQ*_), but not all monocyte simulations produced equilibrium solutions due to the stiffness of the system. In order to determine the parameter ranges that met the dynamical criteria from Table 1 (further detailed in the following section), we simulated chemotherapy for 7 days on each of the non-trivial equilibrium solutions (details of chemotherapy in Additional file 1: Supplement 3). Acceptable solutions met all seven of the dynamical acceptability criteria (*n*_*F*_=177 for neutrophils, *n*_*F*_=1796 for lymphocytes, and *n*_*F*_=1049 for monocytes). Figure 2 depicts the process, and Fig. 3 shows the intersection of all acceptable parameter regions.

### Physiological capabilities of model

Data-consistent simulations not only fit the patient data, but they also meet a number of qualitative and quantitative criteria (summarized in Table 1). These seven capabilities were mostly determined from literature, though we also used data from Dr. Robert Nelson of the Indiana University Simon Cancer Center from patients who received hematopoietic stem cell transplants (HSCT) to determine additional empirical evidence of the patient dynamics (IRB Protocol# 1011009928). Further information on the patient data is included in Additional file 1: Supplement 5. The patient data was used to determine cell concentration bounds, but were not used to validate the time course of patient recovery after transplantation. Thus, a donor infusion was not modeled. The pharmacokinetics and pharmacodynamics of chemotherapy was simulated as described in Additional file 1: Supplement 3, and was implemented as a ‘chemo’ term in the model equations (Additional file 1: Supplement 2). The details of these criteria are below.

1. **Peripheral blood recovers > 80 cells/** ***μ*** **L:** This ensures that the peripheral blood sufficiently recovers after chemotherapy treatment, and the final peripheral blood cell count must be > 80 cells/ *μ*L [58].
2. **Stem cells recovery > 1 cell/** ***μ*** **L:** The final stem cell concentration must be between 1 and 100 cells/ *μ*L. If the stem cell concentration is within the acceptable bounds, the stem cells should also show recovery after chemotherapy occurring [58].
3. **Final dynamic values are within acceptable ranges:** The final uni-lineage peripheral blood cell counts after chemotherapy must be within the upper limits of expected normal peripheral blood cell counts [40, 59]. Neutropenia (low number of neutrophils in the peripheral blood) is very common after undergoing chemotherapy [60], but neutrophilia mostly occurs if another underlying disease exists to cause the high number of neutrophils. We do not want to model neutrophilia during recovery from chemotherapy because that is not a typical response in patients with AML. The values each cell state must be within are depicted in Additional file 1: Supplement 4, Table S6.
4. **Marginal pool is within one order of magnitude of peripheral blood:** The final concentration of the cells in the marginal pool must be within one order of magnitude of the final concentration of the peripheral blood because the size of the marginal pool and peripheral blood compartments are approximately the same [43, 61, 62]. Though a marginal pool can exist for lymphocytes, it is much smaller than that of neutrophils and monocytes [43]. Thus, we do not incorporate a lymphocyte marginal pool in our model.
5. **Cell counts reduce with chemotherapy to <20** ***%*** **of original value:** The concentration of cells remaining at the end of chemotherapy should be less than 20% of the concentration of cells before chemotherapy begins [63].
6. **Recovery overshoot < 12 times value five days after overshoot:** Patients experience lymphopenia, or low white blood cell concentrations, during chemotherapy. After the chemotherapy regimen is completed, patients’ cell counts almost always increase. In some instances, patient cell counts increase and peak above their final homeostatic cell concentration. Here, we define this overshoot level as the maximum concentration of cells after chemotherapy administration is completed. If the overshoot level is more than twelve times the concentration of cells five days after the overshoot (or the concentration of cells at the end of the simulation, whichever comes first), the solution is rejected. This overshoot was the maximum observed in the HSCT patient data from the Simon Cancer Center (Additional file 1: Figure S6 in Supplement 5).
7. **Amplitude of the second peak of oscillations deviates < 18% from the amplitude of the first peak:** Several simulations produced oscillations. Though some small oscillations are reasonable and can occur physiologically, large oscillations are unlikely because they will not maintain normal homeostasis in the body and most patients who are treated for AML do not report large oscillations in cell counts. We manually classified 100 simulations for sufficiently dampened oscillations. We calculated that a simulation was sufficiently dampened if the cell concentration of the second peak value post-chemotherapy was less than an 18% decrease from the first peak post-chemotherapy. Thus, we implemented this cutoff for all of our simulations. An example of a simulation that does not meet this criteria and a simulation that does meet this criteria are shown in Additional file 1: Figure S7 in Supplement 5.

For each uni-lineage parameter search, two criteria were salient in determining which models met the physiological features required based on responses to chemotherapy. The remainder of the criteria are mostly robust in constraining the parameter space. One of the salient criteria was the lack of oscillations for each uni-lineage model. This implies that the need to maintain a steady-state concentration of cells for each of the lineages independently is one of the key constraints in normal hematopoiesis and is consistent with parameters that lead to balanced feedback. The second criteria that largely determined the acceptable uni-lineage models were the final peripheral blood acceptable dynamics of the neutrophil uni-lineage model, stem cell recovery for the lymphocyte uni-lineage model, and sufficient response to chemotherapy for the monocyte uni-lineage model (indicated with asterisks in Fig. 3). This reflects the necessity of maintaining high concentrations of neutrophils in homeostasis and the ability of cells to repopulate cell populations for appropriate recovery. If we relaxed the constraints imposed on the model set by the features of normal physiological dynamics, the acceptable parameter sets would be larger as well.

### Parameter constraints with separatrix method

**Fig. 4:**
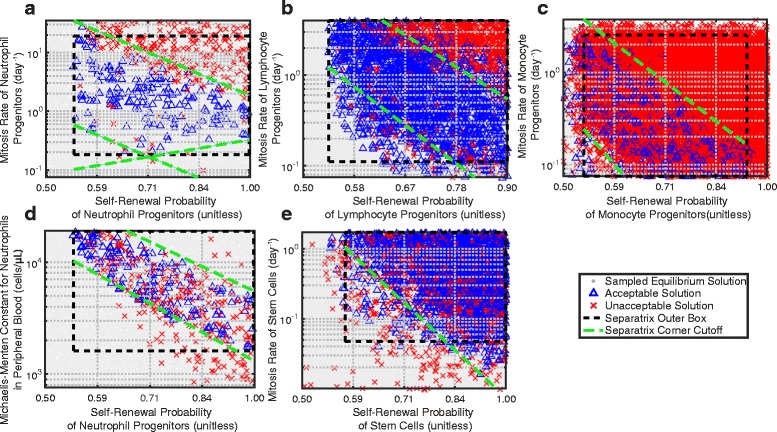
Uni-lineage pair-wise parameter constraints with separatrix method. Five pair-wise parameter relationships from the unilineage models emerged between self-renewal probability and mitosis rate (**a**-**c**, **e**) and the michaelis-menten constant for neutrophils (**d**). For all subfigures, the legend is the same. Gray dots that cover the majority of the background represent all equilibrium solutions that were sampled, but did not meet the final acceptable dynamic values and were zero-equilibrium solutions. Solutions that did not meet the other acceptability criteria are shown with red ‘x’s. Blue triangles represent acceptable parameter sets. We defined a separatrix, which is outlined with a dashed black box that represents the limits of the range of the acceptable parameter space. The dashed green lines show the corners of the separatrix that were removed

We identified several relationships among pairs of uncertain parameters in the models that met the dynamic capabilities of uni-lineage models. This demonstrates parameter relationships that maintain the normal physiological responses to stimuli, and reveals certain physiological processes that are directly related in a specific manner to each other. We discovered that negative exponential relationships exist between pairs of parameters, especially between the fraction of cells that self-renew and the mitosis rates of cells (Fig. 4). This implies that either the mitosis rate or the probability of cell renewal needs to be high for a progenitor cell to sustain its population. If both of these rates are high, however, instability occurs. Traditional methods of separating data in multiple dimensions into groups do not define the bounds of the groups, such as in clustering [64], or do not separate multiple linear separations observed in Fig. 4, such as in support vector machines [65]. A computationally efficient method of separating multiple linear constraints is needed. Thus, to constrain the parameter space based on these relationships, we developed a separatrix method that defined the bounds of the relationships between parameters.

The separatrix method distinguishes between parameter regions of acceptable solutions and regions of unacceptable solutions, and in log-space, this manifests as a clear separatrix. We first constrain to the acceptable range of each parameter (dashed black box in Fig. 4). For each unique pair of uni-lineage parameters, we then divide the parameter range rectangle into 10×10 equally sized bins. If the acceptable parameter set is uniformly distributed in parameter space, every bin has a cutoff of (number of acceptable simulations)/100 acceptable parameter sets. Thus, each of the 100 bins is either acceptable or unacceptable depending on whether more acceptable parameter sets existed in the bin than the cutoff value. We then further constrain this acceptable parameter density by removing corners of the range rectangle that did not maintain a cutoff density of acceptable parameter sets (dashed green lines in Fig. 4). Thus, we found a set of inequalities for each unique pair of parameter sets in the uni-lineage parameterization in log space. We used this separatrix constraint on the parameter search space for multi-lineage parameterization.

The separatrices of each pair-wise parameter relationship confirm parameter relationships from previous works, and we identified additional required relationships in the acceptable solutions. As Getto et al. [38] and Stiehl et al. [52] show, the mitosis rate of stem cells and the probability of self-renewal are inversely related. However, we found that this also extends to all progenitor cells (Fig. 4 a-c). Specifically, a linear relationship emerges between the log of the self-renewal probability and the log of the mitosis rate. Solutions that have larger mitosis rate or self-renewal probabilities produce oscillations (red x’s in Fig. 4), which we constrained against in our dynamical acceptability criteria (Criteria 7 in Table 1). We also found a similar inverse linear relationship between the log of the self-renewal probability of neutrophil progenitors and the log of the homeostatic constant of neutrophils in the peripheral blood; solutions that have lower values of either of these two parameters becomes unacceptable (Fig. 4 d). Overall, this finding means that a specific relationship is maintained between the probability of self-renewal and the mitosis rate for all cells that are capable of self-renewing to ensure that the cell population remains steady. Additionally, due to the high concentration of neutrophils, a constrained feedback mechanism exists between the self-renewal probability of neutrophil progenitors and the cytokines produced by neutrophils in the peripheral blood to maintain appropriate homeostatic concentrations of those cells. Overall, we confirmed the dependence on the mitosis rate and self-renewal probability of stem cells found by other groups (Fig. 4 e), but we also found additional dependencies on these same parameters of progenitor cells, as well as a specific dependence of the cytokines produced by neutrophils in the peripheral blood and the self-renewal probability of neutrophil progenitor cells.

### Multi-lineage acceptable solutions

**Fig. 5:**
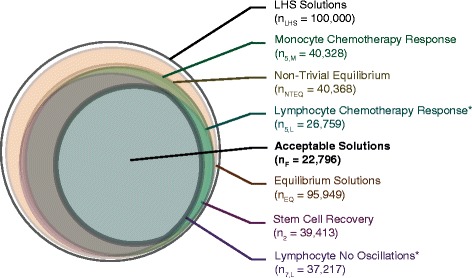
Parameter space constraints for multi-lineage HSC models using dynamic acceptability criteria. Each circle represents the number of simulations that met the acceptability criteria indicated by the corresponding color-coded label. Overlapping circles demonstrate the region in which simulations met multiple criteria

Following analysis of uni-lineage models, we integrated the uni-lineage models into a single multi-lineage model of leukopoiesis and AML to identify the constraints on the rates that regulate normal hematopoietic and leukemic proliferation. To evaluate model performance, we required the complete model to be consistent with all of the constraint data that informed the uni-lineage models. We used the specific parameter relationships gleaned from using separatrices on the uni-lineage models to select a set of models that meet the capabilities of the uni-lineage models. Thus, we simulated 100,000 Latin Hypercube samples of the 44 undetermined parameters in the constrained multi-lineage parameter space (Fig. 5, *n*_*LHS*_). 40,368 parameter sets maintained homeostasis (Fig. 5, *n*_*NTEQ*_); of which, 22,796 simulations with specific parameter sets and initial homeostatic conditions were deemed acceptable simulations using the same seven dynamic criteria from Table 1 (intersection of acceptable parameter spaces in Fig. 5, *n*_*F*_). The lymphocyte uni-lineage models caused the most constraints on the full multi-lineage models, specifically to restrict the oscillations and promote recovery post-chemotherapy in lymphocytes (Fig. 5). This is potentially due to the large concentration of lymphocytes and their rapid selection in the thymus [54] in contrast to their long half-lives in the peripheral blood [25, 55]. Overall, in the final multi-lineage model, the acceptable parameter sets represent 22,796 virtual healthy patients. All of the mean and ranges of the parameters that were varied are in Additional file 1: Supplement 4, Table S8. Correlation coefficients of the initial conditions and parameter values of the multi-lineage model are shown in Additional file 1: Figure S5 in Supplement 4.

**Fig. 6:**
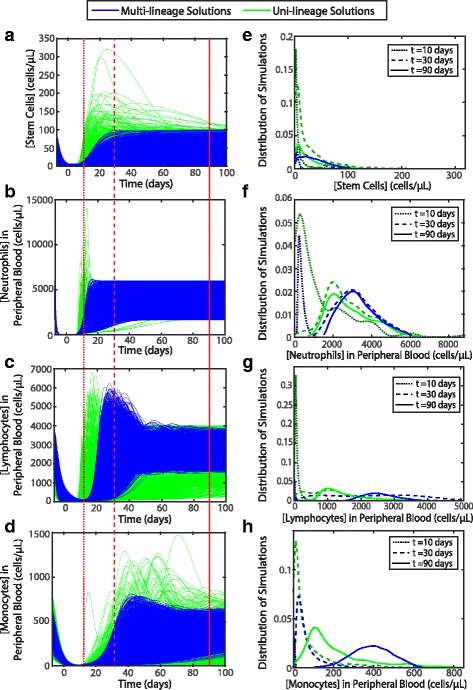
Uni-lineage and multi-lineage simulations comparison. The effects of constraining the uni-lineage parameter space for multi-lineage simulations are shown in (**a**) stem cells, (**b**) neutrophils in the peripheral blood, (**c**) lymphocytes in the peripheral blood, and (**d**) monocytes in the peripheral blood. The distribution of solution concentrations of these cells at three different time points, indicated by red lines in (**a**-**d**), of both multi-lineage and uni-lineage are shown for the same respective states in (**e**-**h**). The time points were chosen to demonstrate the distributions as cell concentrations are recovering from chemotherapy, at the peak of recovery, and homeostasis post-chemotherapy. The uni-lineage acceptable simulations are shown in green and the multi-lineage acceptable simulations are shown in blue in the front of the image. For the distributions of the simulations, the dotted lines are a dark shade of blue or green and represent the earliest time point, the dashed lines represent a time point around the peak of response, and the solid brightest line represents a steady-state time point. The uni-lineage simulations of stem cells are from the lymphocyte uni-lineage. The distributions were scaled such that the area under each curve is 1

A comparison of the simulations of the multi-lineage solutions to the simulations of the uni-lineage solutions is shown in Fig. 6 a-d. In the stem cell and all peripheral blood cell compartments, the uni-lineage models allow for a greater spread of cell dynamics than in the multi-lineage models. Specifically, the overshoot of recovery of cells post-chemotherapy is dampened in the multi-lineage solutions. This means that the combined feedback from cytokines from different lineages has a stabilizing effect. Additionally, in observing the distributions of the concentration of cells of the multi-lineage solutions in comparison to the uni-lineage solutions at specific time points (Fig. 6 e-h), the multi-lineage solutions (blue lines in Fig. 6 e-h) have lower cell concentrations at the early time points (dotted and dashed lines in Fig. 6 e-h) but higher cell concentrations at later time points near homeostasis (solid lines in Fig. 6 e-h). This implies that in order to maintain acceptable cell dynamics in a multi-lineage system, the concentration of cells needs to be at a higher homeostatic concentration than if only considering the uni-lineage model. The overshoot of the lineages in Fig. 6 a-d are all well within patient variation (Additional file 1: Figure S6 in Supplement 5); though, our simulation dynamics may be too constrained in comparison to patient data. Forty out of the 47 patients (85%) in Additional file 1: Figure S6 do not demonstrate neutrophil overshoot, but none of our multi-lineage neutrophil simulations demonstrate this overshoot. We find that constraining the uni-lineage models to multi-lineage data may have constrained the multi-lineage model more than patient overshoot data reflects. We additionally test the robustness of the multiple lineages in the model by perturbing progenitor neutrophils and testing the dynamics before reaching homeostasis on the different lineages when coupled together compared to when decoupled. We found that the multi-lineage model tempers the effects of this perturbation in the neutrophil lineage but causes lymphocyte dynamics to change (Additional file 1: Supplement 6, Figure S8).

### Sensitivity analysis reveals importance of self-renewal probability and cytokines

We carried out a sensitivity analysis on the multi-lineage solutions to determine the most important parameters whose variation most greatly affected output. Using a partial rank correlation coefficient (PRCC) method [66–68], we determined the parameters that were most impactful in determining concentrations of stem cells, neutrophils in the peripheral blood, lymphocytes in the peripheral blood, and monocytes in the peripheral blood. We found that, in general, the probability of self-renewal and the Michaelis-Menten constant that modifies both the rate of self-renewal probability and the movement of mature cells into the peripheral blood were the most important parameters for their respective cell states. This indicates that the probability of a cell to self-renew is very sensitive to changes in the system and can cause very drastic outcomes in the final cell concentration if this rate is affected, which corroborates with findings in Marciniak-Czochra et al. [19]. Additionally, for stem cells, the feedback cytokines from the concentration of cells in the bone marrow and the concentration of stem cells were very important parameters. These constants also modify the probability of self-renewal in the stem cells. Finally, many parameters that are associated with neutrophil homeostasis (self-renewal probability of neutrophil progenitors, mitosis rate, Michaelis-Menten constant, and mobilization rate) all were very important in the stem cell concentration. This is probably due to the large concentration of neutrophils affecting the homeostasis values of stem cells. The PRCC results are in Additional file 1: Supplement 6.

### Representative patient clusters

**Fig. 7:**
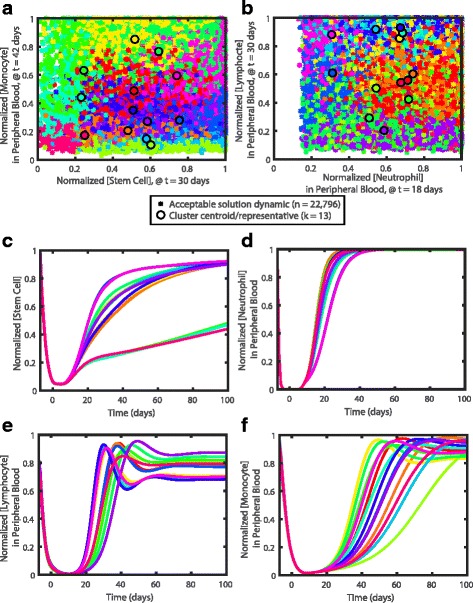
Representative parameter sets. The 22,796 parameter set dynamics of (**a**) normalized stem cell concentration at 30 days after the start of chemotherapy (x-axis) versus the normalized monocyte concentration in the peripheral blood at 42 days after the start of chemotherapy and (**b**) normalized neutrophil concentration in the peripheral blood at 18 days after the start of chemotherapy (x-axis) versus the normalized lymphocyte concentration in the peripheral blood at 30 days after the start of chemotherapy are shown with ‘*’, color-coded according to cluster. The time points plotted were chosen to demonstrate the clustering at times of maximum variation in the dynamics of the particular cell states. The thirteen clusters are each represented with distinct colors, and the centroids of each cluster are shown with a black-outlined circle. To demonstrate the differences in the dynamics of the representatives, the response of (**c**) stem cells, (**d**) neutrophils in the peripheral blood, (**e**) lymphocytes in the peripheral blood, and (**f**) monocytes in the peripheral blood to chemotherapy from day -7 to day 0 are shown normalized to the maximum value of each simulation. Again, each distinct color represents a different cluster centroid, or representative patient, and these colors correspond to the same clusters as in Figures 7 a and b

In order to simulate cancer in a feasible number of patients, we clustered the 22,796 solutions in the normalized dynamic space. This means that we normalized each state in each parameter set simulation by the maximum value simulated. We clustered these normalized simulations across all states and 162 time points (daily from day -7 to day 150 and once every subsequent 30 days until day 300) using k-means, where chemotherapy was applied from day -7 to day 1. We found that 13 clusters maximized the dynamical similarities within a cluster over all states and minimized the overlap in the dynamics across all states between clusters. These 13 clusters represent 13 sub-groups of patients, and the centroid of each of these clusters is the most representative patient for each cluster. For more information on determining the number of clusters, see Additional file 1: Supplement 7. In Fig. 7 a and b, we show how the dynamics are clustered across some of the most varied dynamical regions from Fig. 6. Stem cells group into two distinct behaviors; stem cell concentrations reach homeostasis within two months post-chemotherapy for all but three clusters (Fig. 7 c). Normalized neutrophil dynamics are fairly constrained, and the majority of its variation occurs during the rapid recovery of neutrophils post-chemotherapy (Fig. 7 d). However, the overall dynamics of the normalized clustered representatives demonstrate the variability in both lymphocytic (Fig. 7 e) and monocytic dynamics (Fig. 7 f). In general, the various clusters differ from each other primarily by the normalized monocyte concentration in the peripheral blood post-chemotherapy, overshoot in the concentration of lymphocytes in the peripheral blood, recovery time of neutrophils in the peripheral blood, and the homeostatic concentration of stem cells relative to their initial concentration post-chemotherapy. A correlation analysis did not reflect any significant differences in the independent parameter values amongst the thirteen clusters. However, simulations within individual clusters may have parameter dependencies that are different between clusters. Real patients can potentially be assigned to clusters based on their dynamics following one round of chemotherapy, though outlier patients need to be sufficiently explored to encompass true patient dynamics.

### Modeling leukemia

AML is a derivative of common granulocyte macrophage progenitor cells [3, 69]. Thus, leukemia is modeled parallel to the manner in which monocytes are modeled. We assume that the cancer stem cells self-renew and proliferate at the same rate as monocytic progenitor cells. These then differentiate into mature cancer within the bone marrow, which can mobilize into the peripheral blood. The only difference in the model between leukemic stem cells and monocytes is that the leukemic cells do not maintain homeostasis, so all feedback that regulates self-renewal [18, 70], differentiation, and movement were removed and the cancer cells cannot be recruited back to the tissue. Since we have modeled feedback from various signals, the mutated cancer cell that grows indefinitely is the product of several mutations that cause all feedback to be inhibited, as currently hypothesized in literature [49].

**Fig. 8:**
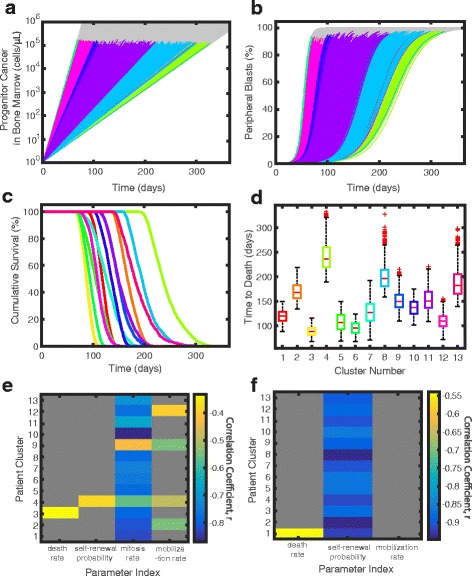
Survival in leukopoiesis. Each of the thirteen clusters is represented with a distinct color in a-d, corresponding to the same colors as in Fig. 7. (**a**) Progenitor cancer stem cells (Mc2) grow over one year in each of the 22,796 simulations. The gray lines represents the cell concentration and time in which patients may die due to overgrowth of cancer cells. (**b**) The percentage of cells in the peripheral blood due to AML in each of the 22,796 simulations. Patients die prior to reaching 100% peripheral blasts, as indicated by gray lines where simulation continued. In (**a**) and (**b**), some cluster simulations are not visible because they are underneath the other simulations. (**c**) The simulated survival curves of each of the thirteen representative clusters of patients are shown assuming the patient dies when either cancer in the bone marrow or the peripheral blood reaches a concentration greater than 3×10^5^ cells/ *μ*L. The cumulative survival is shown as a decimal that represents the fraction of patients that are still alive at the time shown on the x-axis.(**d**) The distribution of the time to death for each cluster is indicated using box plots. Medians of the distribution are shown as red horizontal bars and outliers are shown as red plus signs (+). (**e**) Significant cancer parameter correlation to number of days until death due to bone marrow or peripheral blood density greater than 3×10^5^ (*p*<0.05). (**f**) Significant cancer parameter correlation constrained with a fixed cancer mitosis rate to the number of days until death due to bone marrow density or peripheral blood density greater than 3×10^5^ (*p*<0.05). In figures (**e**) and (**f**), gray boxes represent insignificant correlations, and the correlation values for each of the thirteen clusters are depicted with the color bar

We simulated AML with one cancer stem cell, and allowed the leukemia to clonally accumulate in each of the 22,796 simulated patients (Fig. 8 a), as expected from literature [71]. The variation shows that some patients may survive only a few months after the cancer starts (i.e., pink cluster lines in Fig. 8 a; gray lines indicate death) and some patients may live close to one year without any other intervention (i.e., green cluster lines in Fig. 8 a). In order to determine how long patients in each cluster would live without any treatment, we assumed patients would survive until the concentration of cancer cells in either the peripheral blood or bone marrow was greater than 3×10^5^ cells/ *μ*L (Fig. 8 a-d). This is the theoretical maximal concentration of cells in the bone marrow calculated in Additional file 1: Supplement 3, given the diameter of monocytes and assuming the volume in the bone marrow and peripheral blood are the same. Figure 8 b shows the AML growth as a percentage of the peripheral blood. AML is diagnosed when over 20% of the bone marrow or peripheral blood contains cancer blasts, or immature cancer cells [72], and our model indicates that patients can remain undiagnosed with AML for weeks to months, as previously suggested [73]. The growth of AML is simulated to stop when the theoretical maximum concentration of bone marrow cells is reached. In Fig. 8 b, patients with slower growing cancers die with AML comprising a higher percentage of their peripheral blood. Figs. 8 c-d indicate that patients within some clusters have a much better prognosis than others, and generally correlates with a slower recovery of monocytes post-chemotherapy (corresponding colors in Fig. 7 e).

For a simple validation to ensure that cancer is not growing too slowly in our model, we can compare overall survival times of the simulations without treatments to trials in which patients received low-dose treatments. The majority of clinical patients who are unable to receive traditional chemotherapy and received hydroxyurea as a treatment post-diagnosis with AML died within one year of diagnosis even with favorable stratification [74]. We find the survival time in all of our simulations to be less than four months in after diagnosis of AML >20*%* blasts (Fig. 8 b). Thus, cancer growth fits within an expected bound of patient survival.

To test which parameters correlate with the survival time of patients with AML, we correlated survival time to the four parameters that mediate cancer in our model: (1) the death rate of all cancer cells (dMc), (2) the fraction of cells that self-renew (aMc2), (3) the mitosis rate of cancer progenitor cells (mrMc2), and (4) the mobilization of cancer cells into the peripheral blood (mbMc). For the 22,796 cancer simulations, these parameter values were assumed to be the same as those of monocytes in healthy hematopoiesis. In order to determine which of the parameters that determine survival time the most, we sampled 625 Latin Hypercube Samples for each of the 13 representative patients. For this sampling, we varied the parameters, dMc, aMc2, mrMc2, and mbMc within the ranges shown in Table 2. These bounds were chosen to ensure the death and mobilization rates were slower than those of normal monocytes, but mitosis rates are higher. We computed a correlation of all of the diagnosed cancers in these simulations (bone marrow or peripheral blood blast concentration >20*%* and cancer stem cell concentration >1 cell/ *μ*L) to the number of days of survival. We found that across all clusters, the mitosis rate of cancer was correlated with survival without chemotherapy. The overall negative correlation of -0.7431 (Fig. 8 e) indicates that the higher the mitosis rate, the more likely the patient will die earlier without treatment. We constrained this same search by fixing the mitosis rate. and found that the next most correlated parameter to survival time was the probability of self-renewal of cancer, with an overall correlation of -0.8774 (Fig. 8 f). This result aligns with the inverse relationship found between mitosis rates and self-renewal probability (Fig. 4). Stiehl et al. have similarly found that high proliferation rates and high self-renewal rates can also lead to earlier death [52]. We simulated AML derived from neutrophil progenitors analogously to simulating monocytic AML and confirmed these results (Additional file 1: Supplement 8).

**Table 2:** Cancer parameter bounds

Parameter	Lower bound	Upper bound
*d*_*Mc*_	0.0001*d*_*M*_	*d*_*M*_
*a*_*Mc*2_	0.01*a*_*M*2_	1
*m* *r*_*Mc*2_	0.5*m* *r*_*M*2_	30
*m* *b*_*Mc*_	0.0001*m* *b*_*M*_	*m* *b*_*M*_

Four parameters were varied for determining the most correlated cancer parameter to survival time: the death rate of cancer (*d*_*Mc*_), the self-renewal probability of cancer progenitor cells (*a*_*Mc*2_), the mitosis rate of cancer progenitor cells (*m* *r*_*Mc*2_), and the mobilization rate of cancer stem cells (*m* *b*_*Mc*_). The bounds of these parameters were restricted based on the corresponding parameters of monocytes

## Discussion

When a patient is diagnosed with AML, physicians assess the white blood cell counts of the patient to guide the course of action for treating the leukemia. Many patients are successfully treated with a stem cell or bone marrow transplant from a matched donor. However, for those patients who are unable to receive a transplant, chemotherapy is administered periodically to lower the cancer load on the patient. Generally, a high white blood cell count makes the patient a candidate for chemotherapy. However, it is likely that factors other than the absolute cell count are important for determining a patient’s prognosis. Using a multi-lineage model of the formation of white blood cells, we find that (1) certain physiological rate relationships are necessary to prevent unstable cell population growth and (2) the rate of growth of the cancer is an important prognostic factor in determining the survival time of patients.

We determined that there are specific physiological rates and cell concentrations that are crucial in maintaining homeostasis. As expected, progenitor cells are very important in homeostatic mechanisms. A sensitivity analysis demonstrated that the probability of self-renewal of all progenitor cells and parameters that modify this probability are the most important parameters in hematopoietic dynamics. Other groups have found similar results [19] in the context cancer [75, 76] and specifically AML [77]. Additionally, we found an inverse relationship between the self-renewal probability of stem cells and the mitosis rate of stem cells (Fig. 4 e), corroborating findings of other groups [38, 52]. This relationship between mitosis rate and self-renewal probability also exists for progenitor cells (Fig. 4 a-c), which was not found previously. In general, solutions that have high mitosis rates or self-renewal probabilities lead to oscillations in the cell concentrations. This oscillation is highly undesirable and can occur in diseases such as cyclic neutropenia. Previous work has shown that “re-entry" into the stem cell compartment was found to be one of the factors that control oscillation in cyclic neutropenia [22]. Our work suggests that the analogous self-renewal probability and mitosis rate of the same cell, whether it is the stem cell or another progenitor cell, are both crucial to control to prevent diseases such as cyclic neutropenia. Chemotherapy that targets only one of these rates may not be sufficient in controlling physiological oscillations. Thus, we recommend that the feedback mechanisms that govern the self-renewal probabilities are explored as potential pharmaceutical targets in AML treatment.

We found that peripheral blood concentration levels of neutrophils and lymphocytes are important factors in maintaining homeostasis in our model (Fig. 6), specifically in the multi-lineage context. This was evidenced in three ways. First, when we identified the capabilities of our multi-lineage model, we found that lymphocyte dynamics constrained the range of acceptable solutions for all three lineages (Fig. 5). This is likely due to the high selection rate of lymphocytes in the thymus (dL3, which is modeled as the tissue/bone marrow compartment) [54] and low death rate of lymphocytes in the peripheral blood (dL3pb) [25]; this can lead to fast dynamic changes if not constrained appropriately. Second, the inverse relationship between the self-renewal probability of neutrophil progenitors and the homeostatic term for neutrophil progenitors is a novel relationship in hematopoietic modeling that tightly regulates healthy neutrophil behavior (Fig. 4 d). This means that in order to maintain healthy levels of neutrophils at homeostasis, either the fraction of neutrophil progenitor cells that self-renew has to be low or the feedback mechanism that maintains neutrophil homeostasis has to have a low threshold for turning self-renewal off. Thus, self-renewal is very tightly regulated to ensure that cells do not grow indefinitely. To reduce the cancer load for potential treatment, physicians could target the feedback mechanisms associated with self renewal probability, in particular focusing on cytokine signaling. Third, the large concentration of neutrophils cause the parameters associated with neutrophils to be important parameters in determining the concentration of stem cells, as determined by global sensitivity analysis. We confirmed the importance of neutrophils in relation to lymphocytes in the multi-lineage model by testing the robustness of the multi-lineage model in Additional file 1: Supplement 6. We also found that the neutrophils in our multi-lineage model might be over-constrained by comparing to clinical overshoot data in comparison to the uni-lineage models. Specifically, the neutrophil overshoot of the multi-lineage model only reflected the neutrophil overshoot in 85% of clinical data (Additional file 1: Figure S6 in Supplement 5). Thus, our model encompasses most of the dynamics of the patient population. Furthermore, this reflects the importance of the sensing mechanism of each of these cells to maintain appropriate homeostatic levels. When we modeled cancer, removing these homeostatic terms from our model allowed the cancer to grow indefinitely, as expected. Thus, treatment that targets the feedback receptors on cancer cells can drastically help reduce the cancer load.

Individual patients are likely to develop unique phenotypes of AML. The dynamics and relative ratios of the absolute concentrations of neutrophils, lymphocytes, and monocytes vary widely across the patient population. Furthermore, phenotypic variability of leukemia may depend on the specific source cell within the population of common granulocyte progenitors that first becomes cancerous. Here, we develop a mathematical model that is able to describe patient variability in AML. We find that though cancer will form when homeostatic mechanisms are altered, additional mutations that increase the mitosis rate of cancer will reduce the survival of the patient without intervention (Fig. 8 e). Current chemotherapy regimens inhibit DNA replication, which corresponds to inhibiting the mitosis rate of the cancer [78]. However, we also find that if the mechanism that determines the probability of self-renewal of cancer cells is mutated, then this can be an additional target for pharmaceutical treatments (Fig. 8 f), similar to what has been found in other work [75, 77].

The multi-lineage model developed in this work can be modified to explore mechanisms governing hematopoiesis and leukopoiesis. For example, the model could be adapted to discriminate amongst chemotherapy regimens on simulated patients to identify characteristics of patients that would benefit from certain regimens. Then, this could lead to identification of treatment schedules optimized for individual patients. The patient clusters could be used to stratify real patients by using the dynamics of patients’ responses to chemotherapy to match to a specific simulation and its outcome. Additionally, since we found that the probability of self-renewal is a potential secondary target for chemotherapy, various specific feedback mechanisms could be incorporated to identify the exact cytokine signal that would be the best target for therapy. This could be further used to identify the feedback signals that are most sensitive to cause AML growth. Furthermore, the linear relationship in log-log space between mitosis rates and self-renewal probabilities that was found in our model could be tested experimentally to ensure these relationships exist, though mediating cytokines properly is very difficult. Though we did not find specific relationships amongst the different lineages, the signaling mechanisms that control bone marrow size could also be explored experimentally and compared with the model to regulate overproduction of a particular cell lineage with respect to other lineages. Specifically, GM-CSF is often used to regulate the stem cell production of neutrophils and macrophages [79]. This could be manipulated to identify effects in lymphocyte counts and the production of AML. Overall, the multi-lineage model we present here can be extended to characterize many aspects of hematopoiesis.

The multi-lineage model of hematopoiesis and leukopoiesis developed in this work can be readily adapted and expanded to incorporate many other immunological effects. Various groups have already modeled bone marrow transplantation and its potential complications [31, 33, 34]. Using our model, bone marrow or stem cell transplantation and transplant rejection can be integrated to predict graft rejection, and different lymphocyte sub-types can be added to the model, such as natural killer cells, to model an alternative outcome of transplantation: graft versus host disease. Lymphocytic leukemias can be explored by altering the homeostatic mechanisms of lymphocytes; analogously, myelodysplastic syndromes and minimal residual disease could be further characterized by determining parameter changes that lead to appropriate model behavior. Additionally, a wide array of other immunological diseases or the wide array of patient responses (including a large T-cell repertoire) can be adapted into this multi-lineage model to characterize the complexity of normal function and other immunological diseases. More specifically, future work could characterize the mechanisms that cause spontaneous remission of AML in the presence of bacterial infection [80].

## Conclusions

The multi-lineage mathematical model we have created of hematopoiesis and leukopoiesis can help identify how individuals differ in their white blood cell and leukemia production. This model is useful for several reasons. We have determined crucial parameters and parameter relationships that can be used as potential drug targets for both AML and other potential immunological disorders. For many chemotherapeutic drugs, DNA replication is targeted [78], which aligns with targeting the mitosis rate. However, a combination therapy that also addresses the probability of a cancer cell to self-renew could be potentially helpful for patients whose cancer becomes resistant to the initial therapy. There is no current way to experimentally determine how these different lineages interact and limit each other. Thus, this multi-lineage model is a very powerful tool that can aid in understanding how blood forms normally and misforms into AML in individual patients. In addition, the model captures multiple dynamics that represent specific patient subgroups. By clustering the wide population of individual differences, we find that one advantage of this multi-lineage model is that it can be readily extended to investigate personalization of treatment schedules of individual patients to prolong overall survival.

## Additional file

Additional file 1 Supplementary information is provided as pdf files. Supplementary Information 1. Existing Mathematical Models. Current models that describe a subset of mechanisms described in our model are compared in this section. Supplementary Information 2. Model Equations. All ODE model equations are included, as well as diagrams of the positive and negative feedback included in the model. Supplementary Information 3. Detailed Model Description. The model descriptions explain the mechanisms included in the model. A derivation of the form of asymmetrical self-renewal, the capacity of the bone marrow, and the pharmacodynamics of chemotherapy are also detailed in this section. Supplementary Information 4. Constraint and Parameter Tables. Constraint and parameter value ranges, descriptions, and citations are described in this supplement. Supplementary Information 5. Physiological Capabilities of Model. Qualitative patient data are shown to demonstrate validation of dynamical acceptability criteria. Supplementary Information 6. Sensitivity Analysis. Results from completing PRCC are shown. Supplementary Information 7. Determining Cluster Number. A derivation of the optimal number of clusters to describe the patient variation is explained in this supplement. Supplementary Information 8. Neutrophil-Derived AML. Simulations of AML derived from neutrophil progenitors. (PDF 1502 kb)
