# Supplementary material for: An Integrative multi-lineage model of variation in leukopoiesis and acute myelogenous leukemia
Source: BMC Syst Biol. 2017 Aug 25;11:78. doi: 10.1186/s12918-017-0469-2 (PMC5574150; doi:10.1186/s12918-017-0469-2)
Supplement: Additional file 1 — Supplementary information is provided as pdf files. Supplementary Information 1. Existing Mathematical Models. Current models that describe a subset of mechanisms described in our model are compared in this section. Supplementary Information 2. Model Equations. All ODE model equations are included, as well as diagrams of the positive and negative feedback included in the model. Supplementary Information 3. Detailed Model Description. The model descriptions explain the mechanisms included in the model. A derivation of the form of asymmetrical self-renewal, the capacity of the bone marrow, and the pharmacodynamics of chemotherapy are also detailed in this section. Supplementary Information 4. Constraint and Parameter Tables. Constraint and parameter value ranges, descriptions, and citations are described in this supplement. Supplementary Information 5. Physiological Capabilities of Model. Qualitative patient data are shown to demonstrate validation of dynamical acceptability criteria. Supplementary Information 6. Sensitivity Analysis. Results from completing PRCC are shown. Supplementary Information 7. Determining Cluster Number. A derivation of the optimal number of clusters to describe the patient variation is explained in this supplement. Supplementary Information 8. Neutrophil-Derived AML. Simulations of AML derived from neutrophil progenitors. (PDF 1502 kb) [file 12918_2017_469_MOESM1_ESM.pdf]

Supplementary Information 1: Existing Mathematical Models

Several mathematicals exist currently that describe various processes in hematpoiesis. These models successfully address the physiological processes and problems of their aims. However, we incorporate eight different features into our model to fully explain hematopoiesis and the undeterred growth of leukemia. These features are described in the next section.

Table S1: **Existing HSC Mathematical Models.**

**Model**	**Neutrophils**	**Lymphocytes**	**Monocytes**	**Multi-lineage**	**Cellular Activation**	**Mobilization**	**Marginal Pool**	**Leukemia**
Peng et al., 1996 [1]		✓						
Østby et al., 2003 [2]<br>Østby et al., 2004 [3]<br>Engel et al., 2004 [4]<br>Stiehl et al., 2014 [5]<br>Stiehl et al., 2014 [6]<br>Craig et al., 2016 [7]	✓					✓		
Moore et al., 2004 [8]		✓			✓			
Scholz et al., 2005 [9]<br>Marciniak-Czochra et al., 2013 [10]	✓							
DeConde et al., 2005 [11]		✓			✓			
Takumi et al., 2005 [12]	✓		✓			✓		
Colijn et al., 2005 [13, 14]	✓			✓				
Michor et al., 2005 [15]<br>Haeno et al., 2009 [16]								✓
Marciniak-Czochra et al., 2009 [17]		✓						
Manesso et al., 2012 [18]	✓	✓	✓	✓				
Ho et al., 2013 [19]<br>Lahoz-Beneytez et al., 2016 [20]	✓					✓	✓	
Stiehl et al., 2014 [21]	✓							✓
Székely et al., 2014 [22]		✓	✓	✓				
Integrative Model	✓	✓	✓	✓	✓	✓	✓	✓

References

[1] C. A. Peng, M. R. Koller, and B. O. Palsson. Unilineage model of hematopoiesis predicts self-renewal of stem and progenitor cells based on ex vivo growth data. *Biotechnology and Bioengineering*, 52:24–33, 1996.

[2] I. Ostby, L. S. Rusten, G. Kvalheim, and P. Grottum. A mathematical model for reconstitution of granulopoiesis after high dose chemotherapy with autologous stem cell transplantation. *Journal of Mathematical Biology*, 47:101–136, August 2003.

[3] I. Østby, G. Kvalheim, L. S. Rusten, and P. Grottum. Mathematical modeling of granulocyte reconstitution after high-dose chemotherapy with stem cell support: effect of post-transplant g-csf treatment. *Journal of Theoretical Biology*, 231:69–83, November 2004.

[4] C. Engel, M. Scholz, and M. Loeffler. A computational model of human granulopoiesis to simulate the hematotoxic effects of multicycle polychemotherapy. *Blood*, 104:2323–31, October 2004.

[5] T Stiehl, AD Ho, and A Marciniak-Czochra. The impact of cd34+ cell dose on engraftment after scts: personalized estimates based on mathematical modeling. *Bone marrow transplantation*, 49(1):30–37, 2014.

[6] Thomas Stiehl, Anthony D Ho, and Anna Marciniak-Czochra. Assessing hematopoietic (stem-) cell behavior during regenerative pressure. In *A Systems Biology Approach to Blood*, pages 347–367. Springer, 2014.

[7] Morgan Craig, Antony R Humphries, and Michael C Mackey. A mathematical model of granulopoiesis incorporating the negative feedback dynamics and kinetics of g-csf/neutrophil binding and internalization. *Bulletin of mathematical biology*, 78(12):2304–2357, 2016.

[8] H. Moore and N. K. Li. A mathematical model for chronic myelogenous leukemia (cml) and t cell interaction. *Journal of Theoretical Biology*, 227:513–23, April 2004.

[9] M. Scholz, C. Engel, and M. Loeffler. Modelling human granulopoiesis under poly-chemotherapy with g-csf support. *Journal of Mathematical Biology*, 50:397–439, April 2005.

[10] Anna Marciniak-Czochra and Thomas Stiehl. Mathematical models of hematopoietic reconstitution after stem cell transplantation. *Model Based Parameter Estimation*, 2013.

[11] R. DeConde, P. S. Kim, D. Levy, and P. P. Lee. Post-transplantation dynamics of the immune response to chronic myelogenous leukemia. *Journal of Theoretical Biology*, 236:39–59, September 2005.

[12] K. Takumi, J. Garssen, R. de Jonge, W. de Jong, and A. Havelaar. Release kinetics and cell trafficking in relation to bacterial growth explain the time course of blood neutrophils and monocytes during primary salmonella infection. *International Immunology*, 17:85–93, January 2005.

[13] C. Colijn and M.C. Mackey. A mathematical model of hematopoiesis–i. periodic chronic myelogenous leukemia. *Journal of Theoretical Biology*, 237:117–132, 2005.

[14] C. Colijn and M.C. Mackey. A mathematical model of hematopoiesis–ii. cyclical neutropenia. *Journal of Theoretical Biology*, 237:133–146, 2005.

[15] F. Michor, T. P. Hughes, Y. Iwasa, S. Branford, N. P. Shah, C. L. Sawyers, and M. A. Nowak. Dynamics of chronic myeloid leukaemia. *Nature*, 435:1267–1270, 2005.

[16] H. Haeno, R. L. Levine, D. G. Gilliland, and F. Michor. A progenitor cell origin of myeloid malignancies. *Proceedings of the National Academy of Sciences of the United States of America*, 106:16616–16621, 2009.

[17] Anna Marciniak-Czochra, Thomas Stiehl, A. D. Ho, W. Jager, and W. Wagner. Modeling of asymmetric cell division in hematopoietic stem cells–regulation of self-renewal is essential for efficient repopulation. *Stem Cells Development*, 18:377–85, 2009.

[18] Erica Manesso, José Teles, David Bryder, and Carsten Peterson. Dynamical modelling of haematopoiesis: an integrated view over the system in homeostasis and under perturbation. *The Journal of the Royal Society Interface*, 10, 2012.

[19] Thang Ho, Gilles Clermont, and Robert S. Parker. A model of neutrophil dynamics in response to inflammatory and cancer chemotherapy challenges. *Computers and Chemical Engineering*, 51:187–196, 2013.

[20] Julio Lahoz-Beneytez, Marjet Elemans, Yan Zhang, Raya Ahmed, Arafa Salam, Michael Block, Christoph Niederalt, Becca Asquith, and Derek Macallan. Human neutrophil kinetics: modeling of stable isotope labeling data supports short blood neutrophil half-lives. *Blood*, 127:3431–3438, 2016.

[21] Thomas Stiehl, Natalia Baran, Anthony D. Ho, and Anna Marciniak-Czochra. Clonal selection and therapy resistance in acute leukemias: mathematical modelling explains different proliferation patterns at diagnosis and relapse. *Journal of the Royal Society Interface*, 11, 2014.

[22] Tamás Székely, Kevin Burrage, Marc Mangel, and Michael B. Bonsall. Stochastic dynamics of interacting haematopoietic stem cell niche lineages. *PLOS Computational Biology*, 10, 2014.

Supplementary Information 2: Model Equations

Table S2: **Abbreviations.** Non-subscript state names indicate concentrations of those states.

Category	Acronym/Modifier	Definition
State Names	SC	stem cell
	N	neutrophil
	L	lymphocyte
	M	monocyte
	Ma	macrophage (activated)
	Mc	cancer cells (of monocytic lineage)
	A	apoptotic cells
	chemo	chemotherapy toxicity (function of time)
State Modifiers	H	host bone marrow cells
	bm	bone marrow
	pb	peripheral blood
	mp	marginal pool
	1	stem cell
	2	progenitor cell
	3	mature cell
Parameter Names	p	parameter
	k	feedback constant
	n	normalization factor
Parameter Modifiers	a	self-renewal fraction
	mr	mitotic rate
	mb	mobilization
	f	fraction
	d	death
	sr	self-renewal rate
	dr	differentiation rate
	rec	recruitment
	dm	demargination

Table S3: **Parameter Set Categories.** Seven parameters were determined from the literature. The remainder of the undetermined parameters were constrained using a step-wise approach described in the paper text and Figure 2 of the text. The parameters in the model equations below are highlighted with colors according to the key in this table.

Parameter Category Highlight Color Key
parameters set from literature
multi-lineage parameters
neutrophil uni-lineage parameters
lymphocyte uni-lineage parameters
monocyte uni-lineage parameters
cancer parameters
dynamical parameters

1 Feedback

Figure S1: **Negative Feedback Signals of Model.** The figure notations are the same as in Figure 1 of the text with the addition of inhibition depicted by red bar-headed arrows. The feedback is described in the methods section of the paper.The numbered labels of the feedback are correspond to the numbered constants described in Table S4

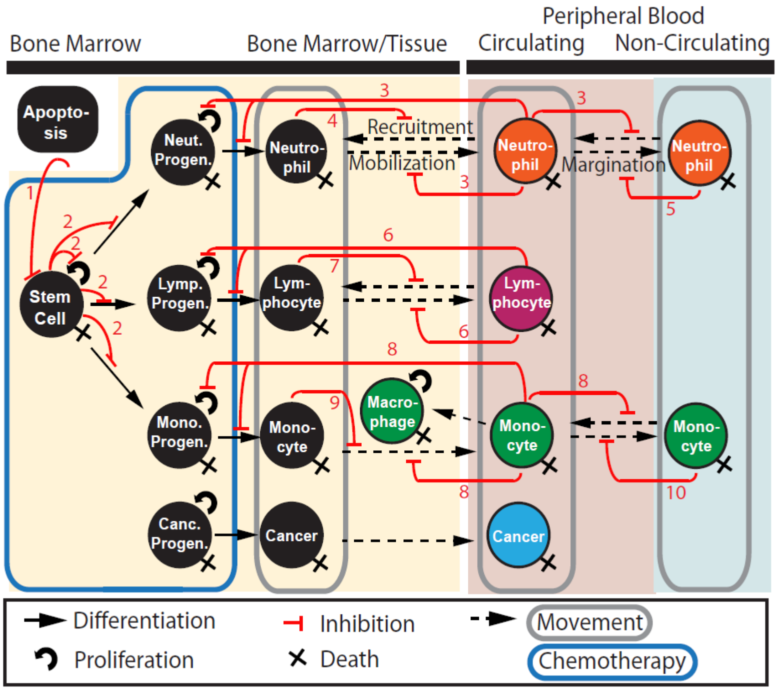

Figure S2: **Positive Feedback Signals of Model.** The figure notations are the same as in Figure 1 of the text with the addition of positive feedback depicted by green dashed arrows. The feedback is described in the methods section of the paper.The numbered labels of the feedback are correspond to the numbered constants described in Table S4

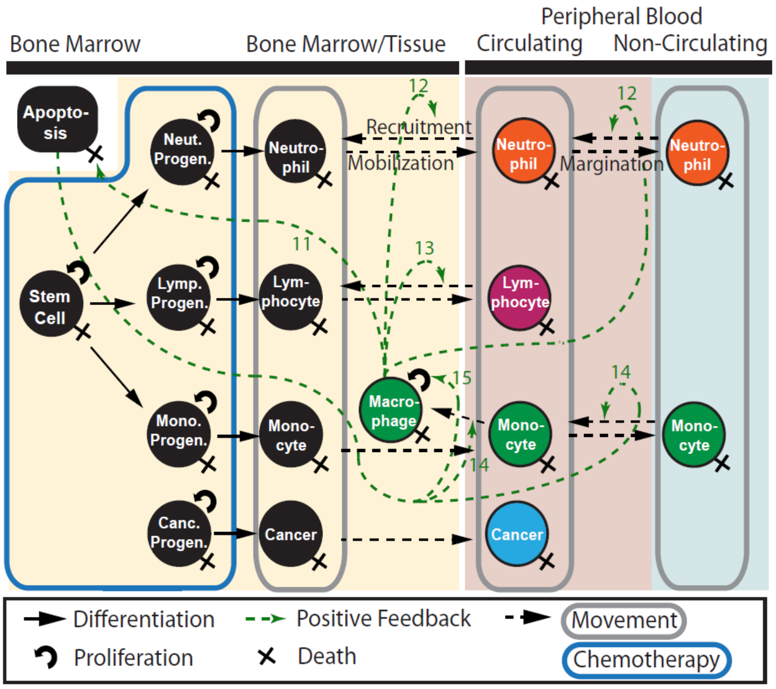

Table S4: **Description of All Feedback Processes.** Each of the feedback processes in our model are described in this table with citations for justification of the feedback process. Signals (cytokines, chemokines, transcription factors, etc.) that approximate these processes are shown in this table. Other factors not listed in this table may also regulate these processes. M-M := Michaelis-Menten; TF := transcription factor; PU.1 and C/EBP α := transcription factors that regulate at the multi-potent progenitor level; EPO:= erythropoietin; GM-CSF : = granulocyte macrophage colony stimulating factor; IL := interleukin; LFA-1 := lymphocyte function-associated antigen 1; SDF-1 := stromal cell-derived factor-1; CXC := chemokine motif "C-X-C"; CC := chemokine motif "C-C" ; LPS := lipopolysaccharide; TGF-β := transforming growth factor; TARC : = T cell-directed CC chemokine thymus and activation-regulated chemokine; Ref. := Reference.

Feedback Label	M-M constant	Signal Approximation	State(s) that generate(s) signal	State(s) that have signal receptor	Process Affected	Neg. or Pos. Feedback	Ref.
1	k_1	PU.1, C/EBP α	all cells in bm	stem cell	self-renewal and differentiation	negative	[1, 2]
2	k_1	EPO	stem cell	stem cell and progenitor cells	self-renewal and differentiation	negative	[3]
3	k_{Npb}	GM-CSF	neut. in the pb	neut. progenitors<br>neut. in the bm<br>neut. in the mp	self-renewal and differentiation<br>mobilization<br>demargination	negative	[4]
4	k_{N3}	IL-17	neut. in the bm	neut. in the pb	recruitment	negative	[5]
5	k_{Nmp}	LFA-1	neut. in the mp	neut. in the pb	margination	negative	[6]
6	k_{Lpb}	SDF-1	lymp. in the pb	lymp. progenitors<br>lymp. in the bm	self-renewal and differentiation<br>mobilization	negative	[7, 8]
7	k_{L3}	LFA-1, IL-8	lymp. in the bm	lymp. in the pb	recruitment	negative	[8]
8	k_{Mpb}	GM-CSF	mono. in the pb	mono. progenitors<br>mono. in the bm<br>mono. in the mp	self-renewal and differentiation<br>mobilization<br>demargination	negative	[4]
9	k_{M3}	CXCR1, CCR2	mono. in the bm	mono. in the pb	recruitment	negative	[9]
10	k_{Nmp}	CXCR1, CCR2	mono. in the mp	mono. in the pb	margination	negative	[9]
11	k_{Ma}	LPS, TGF-β	macrophages	apoptotic debris	clearing of apoptotic debris	positive	[10]
12	k_{Ma}	GM-CSF, CXCR1, CCR2	macrophages	neut. in the pb<br>neut. in the mp	recruitment<br>demargination	positive	[4, 9]
13	k_{Ma}	TARC, CxCL9, CXCL10, CCL17, CCL22, CCL24, IL-12	macrophages	lymp. in the pb	recruitment	positive	[11]
14	k_A	TGF-β	apoptotic debris	mono. in the pb<br>mono. in the mp	recruitment<br>demargination	positive	[12]
15	k_A	TGF-β	apoptotic debris	macrophages	clonal proliferation	positive	[12]

2 Model Equations

A. Cytokine Signal Intensity

$$H = SC1 + N2 + N3 + L2 + L3 + M2 + M3 + Ma + Mc2 + Mc3$$

1. Rate of change for stem cells, SC1

$$\frac{dSC1}{dt} = \begin{array}{ll} +(2a_1 \frac{k_1}{k_1 + \frac{H}{n_{bm}} + \frac{SC1}{n_{SC}}} - 1) p_{mr1} SC1 & \textbf{(Proliferation)} \\ -chemo * SC1 & \textbf{(Chemo Death)} \\ -p_{dp} SC1 & \textbf{(Natural Death)} \end{array}$$

2.1 Neutrophils

2. Rate of change for committed neutrophils, N2

$$\frac{dN2}{dt} = \begin{array}{ll} +2(1 - f_L)(1 - f_M)(1 - a_1 \frac{k_1}{k_1 + \frac{H}{n_{bm}} + \frac{SC1}{n_{SC}}}) p_{mr1} SC1 & (\textbf{Diff.}, \text{ from SC1}) \\ +(2a_{N2} \frac{k_{Npb}}{k_{Npb} + N3pb} - 1) p_{mrN2} N2 & (\textbf{Diff.}, \text{ to N3}) \\ -chemo * N2 & \textbf{(Chemo Death)} \\ -p_{dp} N2 & \textbf{(Natural Death)} \end{array}$$

3. Rate of change for mature neutrophils, N3

$$\frac{dN3}{dt} = \begin{array}{ll} +2(1 - a_{N2} \frac{k_{Npb}}{k_{Npb} + N3pb}) p_{mrN2} N2 & (\textbf{Differentiation}, \text{ from N2}) \\ -\frac{k_{Npb}}{k_{Npb} + N3pb} p_{mbN} N3 & (\textbf{Mobilization}, \text{ to pb}) \\ +\frac{Ma}{k_{Ma} + Ma} \frac{k_{N3}}{k_{N3} + N3} p_{recN} N3pb & (\textbf{Recruitment}, \text{ from pb}) \\ -p_{dN3} N3 & \textbf{(Natural Death)} \end{array}$$

4. Rate of change for mature neutrophils in peripheral blood, N3pb

$$\frac{dN3pb}{dt} = \begin{array}{ll} +\frac{k_{Npb}}{k_{Npb} + N3pb} p_{mbN} N3 & (\textbf{Mobilization}, \text{ from bm}) \\ -\frac{Ma}{k_{Ma} + Ma} \frac{k_{N3}}{k_{N3} + N3} p_{recN} N3pb & (\textbf{Recruitment}, \text{ to bm}) \\ -\frac{k_{Nmp}}{k_{Nmp} + N3mp} p_{recN} N3pb & (\textbf{Margination}, \text{ to mp}) \\ +\frac{Ma}{k_{Ma} + Ma} \frac{k_{Npb}}{k_{Npb} + N3pb} p_{dmN} N3mp & (\textbf{Demargination}, \text{ from mp}) \\ -p_{dN3pb} N3pb & \textbf{(Natural Death)} \end{array}$$

5. Rate of change for mature neutrophils in marginal pool, N3mp

$$\frac{dN3mp}{dt} = \begin{array}{ll} +\frac{k_{Nmp}}{k_{Nmp} + N3mp} p_{recN} N3pb & (\textbf{Margination}, \text{ from pb}) \\ -\frac{Ma}{k_{Ma} + Ma} \frac{k_{Npb}}{k_{Npb} + N3pb} p_{dmN} N3mp & (\textbf{Demargination}, \text{ to pb}) \\ -p_{dN3pb} N3mp & \textbf{(Natural Death)} \end{array}$$

2.2 Lymphocytes

6. Rate of change for committed lymphocytes, L2

$$\frac{dL2}{dt} = \begin{array}{ll} +2 f_L (1 - a_1 \frac{k_1}{k_1 + \frac{H}{n_{bm}} + \frac{SC1}{n_{SC}}}) p_{mr1} SC1 & (\textbf{Differentiation}, \text{ from SC1}) \\ +(2 a_{L2} \frac{k_{Lpb}}{k_{Lpb} + L3pb} - 1) p_{mrL2} L2 & (\textbf{Differentiation}, \text{ to L3}) \\ -chemo * L2 & (\textbf{Chemo Death}) \\ -p_{dp} L2 & (\textbf{Natural Death}) \end{array}$$

7. Rate of change for T cells, L3

$$\frac{dL3}{dt} = \begin{array}{ll} +2(1 - a_{L2} \frac{k_{Lpb}}{k_{Lpb} + L3pb}) p_{mrL2} L2 & (\textbf{Differentiation}, \text{ from L2}) \\ -\frac{k_{Lpb}}{k_{Lpb} + L3pb} p_{mbL} L3 & (\textbf{Mobilization}, \text{ to pb}) \\ +\frac{Ma}{k_{Ma} + Ma} \frac{k_{L3}}{k_{L3} + L3} p_{recL} L3pb & (\textbf{Recruitment}, \text{ from pb}) \\ -p_{dL3} L3 & (\textbf{Natural Death}) \end{array}$$

8. Rate of change for T cells in peripheral blood, L3pb

$$\frac{dL3pb}{dt} = \begin{array}{ll} +\frac{k_{Lpb}}{k_{Lpb} + L3pb} p_{mbL} L3 & (\textbf{Mobilization}, \text{ from bm}) \\ -\frac{Ma}{k_{Ma} + Ma} \frac{k_{L3}}{k_{L3} + L3} p_{recL} L3pb & (\textbf{Recruitment}, \text{ to bm}) \\ -p_{dL3pb} L3pb & (\textbf{Natural Death}) \end{array}$$

2.3 Monocytes

9. Rate of change for committed monocytes, M2

$$\frac{dM2}{dt} = \begin{array}{ll} +2(1 - f_L) f_M (1 - a_1 \frac{k_1}{k_1 + \frac{H}{n_{bm}} + \frac{SC1}{n_{SC}}}) p_{mr1} SC1 & (\textbf{Diff.}, \text{ from SC1}) \\ +(2 a_{M2} \frac{k_{Mpb}}{k_{Mpb} + M3pb} - 1) p_{mrM2} M2 & (\textbf{Diff.}, \text{ to M3}) \\ -chemo * M2 & (\textbf{Chemo Death}) \\ -p_{dp} M2 & (\textbf{Natural Death}) \end{array}$$

10. Rate of change for mature monocytes, M3

$$\frac{dM3}{dt} = \begin{array}{ll} +2(1 - a_{M2} \frac{k_{Mpb}}{k_{Mpb} + M3pb}) p_{mrM2} M2 & (\textbf{Differentiation}, \text{ from M2}) \\ -\frac{k_{Mpb}}{k_{Mpb} + M3pb} p_{mbM} M3 & (\textbf{Mobilization}, \text{ to pb}) \\ -p_{dM3} M3 & (\textbf{Natural Death}) \end{array}$$

11. **Rate of change for mature monocytes in peripheral blood, M3pb**

$$\frac{dM3pb}{dt} = \begin{array}{ll} +\frac{k_{Mpb}}{k_{Mpb}+M3pb} p_{mbM} M3 & (\textbf{Mobilization}, \text{from bm}) \\ -\frac{k_{Mmp}}{k_{Mmp}+M3mp} p_{recM} M3pb & (\textbf{Margination}, \text{to mp}) \\ -\frac{A}{k_A+A} p_{drM3} M3pb & (\textbf{Recruitment}, \text{to Ma}) \\ +\frac{A}{k_A+A} \frac{k_{Mpb}}{k_{Mpb}+M3pb} p_{dmM} M3mp & (\textbf{Demargination}, \text{from mp}) \\ -p_{dM3pb} M3pb & (\textbf{Natural Death}) \end{array}$$

12. **Rate of change for mature monocytes in marginal pool, M3mp**

$$\frac{dM3mp}{dt} = \begin{array}{ll} +\frac{k_{Mmp}}{k_{Mmp}+M3mp} p_{recM} M3pb & (\textbf{Margination}, \text{from pb}) \\ -\frac{A}{k_A+A} \frac{k_{Mpb}}{k_{Mpb}+M3pb} p_{dmM} M3mp & (\textbf{Demargination}, \text{to pb}) \\ -p_{dM3pb} M3mp & (\textbf{Natural Death}) \end{array}$$

2.4 Others

13. **Rate of change for cancer progenitor cells, Mc2**

$$\frac{dM2}{dt} = \begin{array}{ll} +(2a_{Mc2}-1) p_{mrMc2} Mc2 & (\textbf{Differentiation}, \text{to Mc3}) \\ -chemo * Mc2 & (\textbf{Chemo Death}) \\ -p_{dMc} Mc2 & (\textbf{Natural Death}) \end{array}$$

14. **Rate of change for cancer blasts, Mc3**

$$\frac{dM3}{dt} = \begin{array}{ll} +2(1-a_{Mc2}) p_{mrMc2} Mc2 & (\textbf{Differentiation}, \text{from Mc2}) \\ -p_{mbMc} Mc3 & (\textbf{Mobilization}, \text{to pb}) \\ -p_{dMc} Mc3 & (\textbf{Natural Death}) \end{array}$$

15. **Rate of change for cancer blasts in the peripheral blood, Mc3pb**

$$\frac{dM3pb}{dt} = \begin{array}{ll} +p_{mbMc} Mc3 & (\textbf{Mobilization}, \text{from bm}) \\ -p_{dMc} Mc3pb & (\textbf{Natural Death}) \end{array}$$

16. **Rate of change for activated macrophages, Ma**

$$\frac{dMa}{dt} = \begin{array}{ll} +\frac{A}{k_A+A} p_{srMa} Ma & (\textbf{Proliferation}, \text{Induced}) \\ +\frac{A}{k_A+A} p_{drM3} M3pb & (\textbf{Recruitment}, \text{from M3pb}) \\ -chemo * Ma & (\textbf{Chemo Death}) \\ -p_{dMa} Ma & (\textbf{Natural Death}) \end{array}$$

17. **Rate of change of cellular debris and apoptotic cells, A**

$$\frac{dA}{dt} = \begin{array}{ll} +chemo * (SC1 + N2 + L2 + M2 + Ma + Mc2) & (\textbf{Chemo Death}) \\ -\frac{Ma}{k_{Ma}+Ma} p_{dMaR} A & (\textbf{Ma Cleanup}) \end{array}$$

References

[1] Edward W Scott, M Celeste Simon, John Anastasi, and Harinder Singh. Requirement of transcription factor pu. 1 in the development of multiple hematopoietic lineages. *Science*, 265(5178):1573–1578, 1994.

[2] Laura T Smith, S Hohaus, DA Gonzalez, SE Dziennis, and DG Tenen. Pu. 1 (spi-1) and c/ebp alpha regulate the granulocyte colony-stimulating factor receptor promoter in myeloid cells. *Blood*, 88(4):1234–1247, 1996.

[3] Cheng C. Zhang and Harvey F. Lodish. Cytokines regulating hematopoietic stem cell function. *Curr Opin Hematol*, 15:307311, 2008.

[4] Donald Metcalf. Hematopoietic cytokines. *Blood*, 111(2):485–491, 2008.

[5] Martti Laan, Zhi-Hua Cui, Hiroshi Hoshino, Jan Lötvall, Margareta Sjöstrand, Dieter C Gruenert, Bengt-Eric Skoogh, and Anders Lindén. Neutrophil recruitment by human il-17 via cxc chemokine release in the airways. *The Journal of Immunology*, 162(4):2347–2352, 1999.

[6] A. Klonz, K. Wonigeit, R. Pabst, and J. Westermann. The marginal blood pool of the rat contains not only granulocytes, but also lymphocytes, nk-cells and monocytes: a second intravascular compartment, its cellular composition, adhesion molecule expression and interaction with the peripheral blood pool. *Scandanavian Journal of Immunology*, 44:461–9, 1996.

[7] Alessandro Aiuti, IJ Webb, C Bleul, T Springer, and JC Gutierrez-Ramos. The chemokine sdf-1 is a chemoattractant for human cd34+ hematopoietic progenitor cells and provides a new mechanism to explain the mobilization of cd34+ progenitors to peripheral blood. *Journal of Experimental Medicine*, 185(1):111–120, 1997.

[8] David H Adams and Andrew Rlloyd. Chemokines: leucocyte recruitment and activation cytokines. *The Lancet*, 349(9050):490–495, 1997.

[9] Chao Shi and Eric G Pamer. Monocyte recruitment during infection and inflammation. *Nature Reviews Immunology*, 11(11):762–774, 2011.

[10] Valerie A Fadok, Donna L Bratton, Anatole Konowal, Peter W Freed, Jay Y Westcott, and Peter M Henson. Macrophages that have ingested apoptotic cells in vitro inhibit proinflammatory cytokine production through autocrine/paracrine mechanisms involving tgf-beta, pge2, and paf. *Journal of Clinical Investigation*, 101(4):890, 1998.

[11] Toshio Imai, Morio Nagira, Shin Takagi, Mayumi Kakizaki, Miyuki Nishimura, Jianbin Wang, Patrick W Gray, Kouji Matsushima, and Osamu Yoshie. Selective recruitment of ccr4-bearing th2 cells toward antigen-presenting cells by the cc chemokines thymus and activation-regulated chemokine and macrophage-derived chemokine. *International immunology*, 11(1):81–88, 1999.

[12] Valerie A Fadok, Donna L Bratton, Lindsay Guthrie, and Peter M Henson. Differential effects of apoptotic versus lysed cells on macrophage production of cytokines: role of proteases. *The Journal of Immunology*, 166(11):6847–6854, 2001.

Supplementary Information 3: Detailed Model Description

1 Mechanisms of Leukopoiesis

The justification for each of the eight processes included in our model is explained in the following table.

Table S5: **States and mechanisms of leukopoiesis.**

**State or Mechanism**	**Reasoning for Model Requirement**
Neutrophils	Represents the majority of white blood cell count and provides a mechanism of G-CSF control [1]; typically largest white blood cell population [2, 3]; easy to validate due to being commonly measured
Lymphocytes	Second most common white blood cell [3]; potential to expand the model for a mechanism for graft-versus-leukemia or graft-versus-host-disease [4–7]
Monocytes	Clears apoptotic debris post-chemotherapy [8]
Multi-lineage	Exhibits the dynamical differences amongst cell types; Demonstrates lineage dominance and cell-cell interactions (See Supplement 6: "Robustness of Multi-lineage Model")
Cellular Activation	Differentiates between latent mature leukocytes and activated leukocytes [1, 9–12]
Mobilization	Incorporates states with easily validatable peripheral blood data; Provides a mechanism of transport [1, 9–11, 13]
Marginal Pool	Demonstrates rapid demargination of neutrophils and monocytes into peripheral blood after initial chemotherapy or corticosteroid doses [14]
Leukemia	Represents the leukemic variability in patients; Validates the effectiveness of the treatment variables on patients

2 Self-Renewal

Self-renewal and differentiation rates are modeled using the model proposed by Marciniak-Czochra [15]. They are detailed below.

Figure S3: **Stem cell self-renewal and differentiation.**

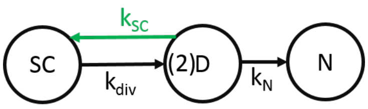

A stem cell, SC, has the potential to self-renew or differentiate into a mature cell, N, as shown above in Figure S3. When stem cells make the decision to create two daughter cells, a quasi-equilibrium state is maintained, D. These daughter cells have the option of remaining as stem cells or becoming mature cells at some rate. Thus, when excluding terms other than the self-renewal and division rates, the mitosis term can be simplified as such:

$$\frac{dSC}{dt} = k_{SC}D - k_{div}SC \tag{1}$$

$$\frac{dD}{dt} = 2k_{div}SC - k_{SC}D - k_N D \tag{2}$$

$$\frac{dN}{dt} = k_N D \tag{3}$$

Quasi-equilibrium is maintained in the daughter state, so setting Equation 2 to 0, we can solve to get

$$D = 2\frac{k_{div}}{k_{SC}+k_N}SC \tag{4}$$

$$\alpha = \frac{k_{SC}}{k_{SC}+k_N} \tag{5}$$

where α is a term that represents the fraction of cells that will self-renew when undergoing mitosis. Thus, we can simplify Equations 1 and 3 to

$$\frac{dSC}{dt} = (2\alpha - 1)k_{div}SC \tag{6}$$

$$\frac{dN}{dt} = 2(1-\alpha)k_{div}SC \tag{7}$$

In this formulation, α must remain between 0.5 and 1 in order to ensure that $\frac{dSC}{dt}$ and $\frac{dN}{dt}$ do not go negative due to the mitosis term (Equation 8). For the remainder of the paper, we refer to k_div as a mitosis rate, mr.

As HSCs progress in their differentiation, they lose their ability to self-renew. In Marciniak-Czochra et al. [16], this theory is maintained by ensuring that α, the fraction of cells that self-renew gets smaller as cells mature. Thus, progenitor cells will have smaller αs than stem cells (Equation 9). Additionally, the mitosis rates of stem cells are very small, and the mitosis rates of more mature cells should be faster (Equation 10).

$$0.5 < \alpha < 1 \tag{8}$$

$$\alpha_{-1} > \alpha_0 \tag{9}$$

$$mr_{-1} < mr_0 \tag{10}$$

Stem cells can differentiate into many types of progenitors. In order to maintain mass balance, we have modeled that a fraction of these cells become lymphocyte progenitors, f_L, a fraction of the non lymphocytes become monocyte progenitors, $(1-f_L)f_M$, and the remainder of the cells become neutrophil progenitors, $(1-f_L)(1-f_M)$.These progenitor cells have the capability of producing mature cells of their specific lineages [17, 18].

3 Homeostatic Feedback of Stem Cells: Capacity of Bone Marrow

To ensure that the concentration of the marrow cells do not exceed the capacity of the marrow, we include a homeostatic feedback term to the stem cells regeneration capability. This term is a Hill term with a constant (k_1) that modulates the concentration of cells in the bone marrow (H) and the number of stem cells in the bone marrow (SC_1), divided by constants (n_{bm} and n_{sc}) to bring these terms to the same scale. We assume that the maximum concentration of cells in the bone marrow is the concentration of a single cell over its own volume. We assume that the concentration of cells in the bone marrow will not exceed the concentration of one cell per volume of one normal monocyte with a diameter of 20 μm [19], as monocytes are the largest inactivated type of white blood cell in our model.

$$\text{Volume of monocyte} = 4/3\pi(10\mu m)^3 = 4.18 \times 10^3 \mu m^3 \tag{11}$$

Thus, we limit n_{bm} to between the concentration of one red blood cell over its own volume, with a diameter of 7 μm [20] and the concentration of one monocyte over its own volume because we assume all of the white blood cells are between the size of a red blood cell and a monocyte. We calculate the approximate maximum concentration of cells in the bone marrow to be, converted to cells per microliter (Equation 12):

$$\frac{\text{1 monocyte}}{\text{volume of 1 monocyte}} = \frac{\text{1 cell}}{4.18 \times 10^3 \mu m^3} \times \frac{1\mu m^3}{1 \times 10^{-9}\mu L} = 2.39 \times 10^5 cells/\mu L \tag{12}$$

Thus, we started our search for n_{bm} at 3×10^5, with a maximum of 5.5×10^6, if the cells have the diameter of a red blood cell. For simplicity, we set our maximum concentration limit to 3×10^5 cells/μL to characterize patient death.

4 Chemotherapy

In order to elucidate the variation in dynamics of individual patients in response to input and to attain the range of parameters that lead to these dynamics, we incorporate a general term for cell loss due to chemotherapy. In this mathematical model, chemotherapy causes death of the stem cells and progenitor cells at a value that mimics pharmacodynamic (PD) effects. The overall PD effect is modeled to be a scalar greater than zero. This scalar is multiplied to a general decay term for all cells that are affected by chemotherapy, and these cells are moved into the apoptotic state, which are cleared by macrophages, as described in Supplement 2.

The PD effect of one treatment cycle is modeled as a combination of drugs that are administered daily for seven days. The drug administration is a product of two exponentials that takes into account both the response time, t_r, and the duration of action, t_d (Equation 13).

$$PD_i(t) = w_i \times dose_i \times (1 - e^{\frac{-(t-t_i)}{t_r}})e^{\frac{-(t-t_i)}{t_d}} \tag{13}$$

where PD_i is the pharmacodynamic effect of one drug, i, and t_i is the time of drug administration for drug i, w_i is a constant to modulate the effect of drug i, and $dose$ is the dose of drug i. The effects of the administration of each drug are summed together for the overall PD effect of the drugs.

For the purpose of simulating dynamics in virtual 'patients' with unique parameter sets, we simulate the effects of the combination of drugs, cyclophosphamide for the first two days, and fludarabine for the 5 subsequent days. The t_r and t_d for cyclophosphamide are 0.1/day and 0.31/day, respectively [21], and the t_r and t_d for fludarabine are 0.125/day [22] and 0.54/day [23], respectively. Thus, a stronger pharmacodynamic effect occurs for the first two days of chemotherapy compared to the last five days of chemotherapy. We do not fit for chemotherapeutic precision, so we assume that the $w_i \times dose_i$ for each drug is 1. For the purposes of this paper, chemotherapy is applied on day -7 through day -1.

References

[1] Donald Metcalf. Hematopoietic cytokines. *Blood*, 111(2):485–491, 2008.

[2] C. Wayne Smith. Production, distribution, and fate of neutrophils. In K. Kaushansky, M. A. Lichtman, J. T. Prchal, M. M. Levi, O.W. Press, L. J. Burns, and M. Caligiuri, editors, *Williams Hematology*, chapter 61. McGraw-Hill, New York, NY, 2015.

[3] Tao Le and Vikas Bhushan, editors. *First Aid for the USMLE Step 1 2012*. McGraw-Hill Education, New York, 2012.

[4] Erica Manesso, José Teles, David Bryder, and Carsten Peterson. Dynamical modelling of haematopoiesis: an integrated view over the system in homeostasis and under perturbation. *The Journal of the Royal Society Interface*, 10, 2012.

[5] F. Baron, M. B. Maris, B. M. Sandmaier, B. E. Storer, M. Sorror, R. Diaconescu, A. E. Woolfrey, T. R. Chauncey, M. E. Flowers, M. Mielcarek, et al. Graft-versus-tumor effects after allogeneic hematopoietic cell transplantation with nonmyeloablative conditioning. *Journal of Clinical Oncology*, 23:1993–2003, 2005.

[6] Georg A. Holländer, Barbara Widmer, and Steven J. Burakoff. loss of normal thymic repertoire selection and persistence of autoreactive t cells in graft vs host disease. *Journal of Immunology*, 152:1609–1617, 1994.

[7] M. Mielcarek, P. J. Martin, W. Leisenring, M. E. Flowers, D. G. Maloney, B. M. Sandmaier, M. B. Maris, and R. Storb. Graft-versus-host disease after nonmyeloablative versus conventional hematopoietic stem cell transplantation. *Blood*, 102:756–762, 2003.

[8] Valerie A Fadok, Donna L Bratton, Anatole Konowal, Peter W Freed, Jay Y Westcott, and Peter M Henson. Macrophages that have ingested apoptotic cells in vitro inhibit proinflammatory cytokine production through autocrine/paracrine mechanisms involving tgf-beta, pge2, and paf. *Journal of Clinical Investigation*, 101(4):890, 1998.

[9] Martti Laan, Zhi-Hua Cui, Hiroshi Hoshino, Jan Lötvall, Margareta Sjöstrand, Dieter C Gruenert, Bengt-Eric Skoogh, and Anders Lindén. Neutrophil recruitment by human il-17 via cxc chemokine release in the airways. *The Journal of Immunology*, 162(4):2347–2352, 1999.

[10] David H Adams and Andrew Rlloyd. Chemokines: leucocyte recruitment and activation cytokines. *The Lancet*, 349(9050):490–495, 1997.

[11] Chao Shi and Eric G Pamer. Monocyte recruitment during infection and inflammation. *Nature Reviews Immunology*, 11(11):762–774, 2011.

[12] Toshio Imai, Morio Nagira, Shin Takagi, Mayumi Kakizaki, Miyuki Nishimura, Jianbin Wang, Patrick W Gray, Kouji Matsushima, and Osamu Yoshie. Selective recruitment of ccr4-bearing th2 cells toward antigen-presenting cells by the cc chemokines thymus and activation-regulated chemokine and macrophage-derived chemokine. *International immunology*, 11(1):81–88, 1999.

[13] Alessandro Aiuti, IJ Webb, C Bleul, T Springer, and JC Gutierrez-Ramos. The chemokine sdf-1 is a chemoattractant for human cd34+ hematopoietic progenitor cells and provides a new mechanism to explain the mobilization of cd34+ progenitors to peripheral blood. *Journal of Experimental Medicine*, 185(1):111–120, 1997.

[14] A. Klonz, K. Wonigeit, R. Pabst, and J. Westermann. The marginal blood pool of the rat contains not only granulocytes, but also lymphocytes, nk-cells and monocytes: a second intravascular compartment, its cellular composition, adhesion molecule expression and interaction with the peripheral blood pool. *Scandanavian Journal of Immunology*, 44:461–9, 1996.

[15] Anna Marciniak-Czochra, Thomas Stiehl, A. D. Ho, W. Jager, and W. Wagner. Modeling of asymmetric cell division in hematopoietic stem cells–regulation of self-renewal is essential for efficient repopulation. *Stem Cells Development*, 18:377–85, 2009.

[16] Anna Marciniak-Czochra and Thomas Stiehl. Mathematical models of hematopoietic reconstitution after stem cell transplantation. *Model Based Parameter Estimation*, 2013.

[17] S. J. Morrison, N. Uchida, and I. L. Weissman. The biology of hematopoietic stem cells. *Annual Review of Cell and Developmental Biology*, 11:35–71, 1995.

[18] T. Reya, S. J. Morrison, M. F. Clarke, and I. L. Weissman. Stem cells, cancer, and cancer stem cells. *Nature*, 414:105–111, 2001.

[19] Robert I. Handin, Samuel E. Lux, and Thomas P. Stossel. In *Blood: Principles and Practice of Hematology*, page 471. Lippincott Williams and Wilkins, Philadelphia, 2003.

[20] Mary Louise Turgeon. In *Clinical Hematology: Theory and Procedures*, page 100. Lippincott Williams and Wilkins, 2004.

[21] Cytoxan clinical pharmacology. `http://www.rxlist.com/cytoxan-drug/clinical-pharmacology.htm`, June 2013. Accessed: 2016-08-22.

[22] Varsha Gandhi and William Plunkett. Cellular and clinical pharmacology of fludarabine. *Clinical Pharmacokinetics*, 41:93–103, February 2002.

[23] L. Malspeis, M.R. Grever, A.E. Staubus, and D. Young. Pharmacokinetics of 2-f-ara-a (9-beta-d-arabinofuranosyl-2-fluoroadenine) in cancer patients during the phase i clinical investigation of fludarabine phosphate. *Seminars in Oncology*, 17:18–32, 1990.

Supplementary Information 4: Constraint and Parameter Tables

1 Model Validation

In order to ensure that our model maintained normal homeostatic cell concentrations, we set one of our healthy dynamic criteria to fit concentrations found from literature (below).

Table S6: **Expected Ranges of Cell State Concentrations.**

State	Abbreviation	Expected Value/Range (cells/μL of compartment)	Reference
States representing cells in bone marrow compartment			
Hematopoietic Stem Cells	SC1	[1×10^0 - 1×10^2]	[1–4]
Naive Neutrophils	N2	[4.2×10^4 - 6.7×10^4]	[5, 6]
Mature Neutrophils	N3	[5.1×10^4 - 3.9×10^5]	[5]
Mature Lymphocytes	L3	6.8×10^4	[6]
Mature Monocytes	M3	1.3×10^3	[6]
States representing cells in peripheral blood compartment			
Mature Neutrophils	N3pb	[1×10^3 - 6×10^3]	[2, 5, 7]
Mature Lymphocytes	L3pb	[8×10^2 - 4×10^3]	[7]
Mature Monocytes	M3pb	[8×10^1 - 8×10^2]	[7]
States representing cells in marginal pool compartment			
Mature Neutrophils	N3mp	[1×10^3 - 6×10^3]	[2, 5, 7]
Mature Monocytes	M3mp	[8×10^1 - 8×10^2]	[5, 7]

2 Parameter Tables

In this section, the values of the parameters and a description of their mechanism are shown. First, the values of the parameters set from literature are shown in Table S7. Next, the general range (25th percentile to 75th percentile) of all parameter values that were sampled using Latin Hypercube Sampling are shown, along with the median value (Table S8. Finally, a comparison of the parameters that are analagous to each other for each of the uni-lineages are shown (Figure S4).

Table S7: **Healthy hematopoiesis parameters set in literature.**

**Parameter**	Value	**Units**	**Description**	**Reference**
d_p	0.0083	day^{-1}	death rate of progenitor cells	[8]
d_N	2.182	day^{-1}	death rate of mature neutrophils	[9]
d_{L3}	0.98	day^{-1}	death rate of mature lymphocytes (thymus selection)	[10]
d_{Lpb}	0.04	day^{-1}	death rate of lymphocytes in the peripheral blood	[11]
d_{M3}	0.21	day^{-1}	death rate of mature monocytes	[12, 13]
d_{Mpb}	0.5	day^{-1}	death rate of monocytes in the peripheral blood	[12]
d_{Ma}	0.0125	day^{-1}	death rate of activated macrophages	[1]

Table S8: **Healthy Hematopoiesis Parameter Values.**

**Parameter**	**25% quartile**	**median**	**75% quartile**	**Units**	**Description**
**Parameters Set in Multi-lineage Model (n = 22,796)**					
a_1	0.8627	0.9336	0.9966	–	self-renewal fraction of stem cells
mr_1	0.2438	0.3147	0.3973	day^{-1}	maximal proliferation rate of stem cells
k_1	1.6534	2.1607	2.7260	–	equilibrium constant: all cells in model
n_{bm}	7.6608e5	9.9926e5	1.2422e6	cells μL^{-1}	normalizing constant: all bone marrow states
n_{sc}	28.8346	35.3980	44.5908	cells μL^{-1}	normalizing constant: stem cell states
f_L	0.1153	0.2445	0.5006	–	fraction of stem cells that differentiate into lymphocytes
f_M	0.0640	0.1588	0.4011	–	fraction of non-lymphocytes that differentiate into monocytes
**Parameters Set in Neutrophil Uni-lineage Model (n = 22,796)**					
a_{N2}	0.6357	0.6828	0.7433	–	self-renewal fraction of neutrophil progenitors
mr_{N2}	0.7644	0.9912	1.2632	day^{-1}	maximal proliferation rate of neutrophil progenitors
mb_N	0.0483	0.0621	0.0792	day^{-1}	mobilization rate of neutrophils
rec_N	0.7019	1.1702	2.0157	day^{-1}	recruitment rate of neutrophils
$homeo_N$	0.3143	0.5608	0.9881	day^{-1}	homeostatic rate of neutrophils
k_{N3}	7.6169e4	1.0885e5	1.5610e5	cells μL^{-1}	equilibrium constant: mature neutrophils
k_{Npb}	6.9996e3	8.9073e3	1.1647e4	cells μL^{-1}	equilibrium constant: neutrophils in peripheral blood
k_{Nmp}	2.9808e3	4.6697e3	7.3909e3	cells μL^{-1}	equilibrium constant: neutrophils in marginal pool
**Parameters Set in Lymphocyte Uni-lineage Model (n = 22,796)**					
a_{L2}	0.6733	0.7041	0.7384	–	self-renewal fraction of lymphocyte progenitors
mr_{L2}	0.5356	0.6972	0.9155	day^{-1}	maximal proliferation rate of lymphocyte progenitors
mb_L	0.0305	0.0403	0.0549	day^{-1}	mobilization rate of lymphocytes
rec_L	1.0069	1.4297	1.9090	day^{-1}	recruitment rate of lymphocytes
k_{L3}	4.2672e4	8.0415e4	1.4583e5	cells μL^{-1}	equilibrium constant: mature lymphocytes
k_{Lpb}	4.9637e3	6.0467e3	7.4015e3	cells μL^{-1}	equilibrium constant: lymphocytes in peripheral blood
**Parameters Set in Monocyte Uni-lineage Model (n = 22,796)**					
a_{M2}	0.6439	0.6937	0.7384	–	self-renewal fraction of monocyte progenitors
mr_{M2}	0.2118	0.2824	0.3708	day^{-1}	maximal proliferation rate of monocyte progenitors
mb_M	0.0444	0.0590	0.0784	day^{-1}	mobilization rate of neutrophils
rec_M	2.7515	4.3339	6.8259	day^{-1}	recruitment rate of monocytes
$homeo_M$	0.1390	0.2077	0.3119	day^{-1}	homeostatic rate of monocytes
k_{M3}	671.2392	1.1148e3	1.8607e3	cells μL^{-1}	equilibrium constant: mature monocytes
k_{Mpb}	1.0490e3	1.2780e3	1.5329e3	cells μL^{-1}	equilibrium constant: monocytes in peripheral blood
k_{Mmp}	180.2485	303.8643	517.8289	cells μL^{-1}	equilibrium constant: monocytes in marginal pool

Table S9 shows parameters that dictate the ability of macrophages to clean up apoptotic debris and to recruit other cells to assist in clearing or attacking pathogens. For those parameters not found in literature, these parameters were nominally set to illicit minimal responses from macrophages. However, this aspect of the model can be explored further for studies that characterize macrophage behavior.

Table S9: **Dynamical parameters.**

**Parameter**	Value	**Units**	**Description**	**Reference**
dr_{M3}	0.28	day^{-1}	activation rate of monocytes to macrophages	[1]
sr_{Ma}	12	day^{-1}	activated macrophage proliferation rate	[14]
k_A	1×10^7	cells μL^{-1}	saturation term for apoptotic cells to activate macrophages	set larger than bone marrow conc. (Supplement 3)
d_{MaR}	1	day^{-1}	removal rate of apoptotic cells by activated macrophages	set to 1 for dynamical studies
k_{Ma}	1	cells μL^{-1}	Michaelis-Menten constant for macrophage recruitment of other cells	set to 1 for dynamical studies

Several observations are made from Figure S4. The self-renewal probability of stem cells is much higher than progenitor cells, and the mitosis rate of stem cells is lower than that of the other progenitor cells, as expected. Additionally, the recruitment rate of monocytes is much larger than the recruitment rate of the other cells, potentially due to the mechanism of forming monocytes for clearing apoptotic debris. Finally, the Michaelis-Menten constants associated with neutrophils and lymphocytes are much larger than that of monocytes, potentially due to the much larger concentrations that exist for neutrophils and lymphocytes than for monocytes.

Figure S4: **Comparison of Analagous Parameter Ranges from Uni-lineage Models.** Abbreviations for these parameters can be found in Table S2 in Supplement 2. a := probability of self-renewal; mr := mitosis rate; mb := mobilization rate; rec := recruitment rate; k := Michalis-Menten constant; '1' := stem cell; '2' := progenitor cell; N := neutrophil; L := lymphocyte; M := monocyte; pb := peripheral blood

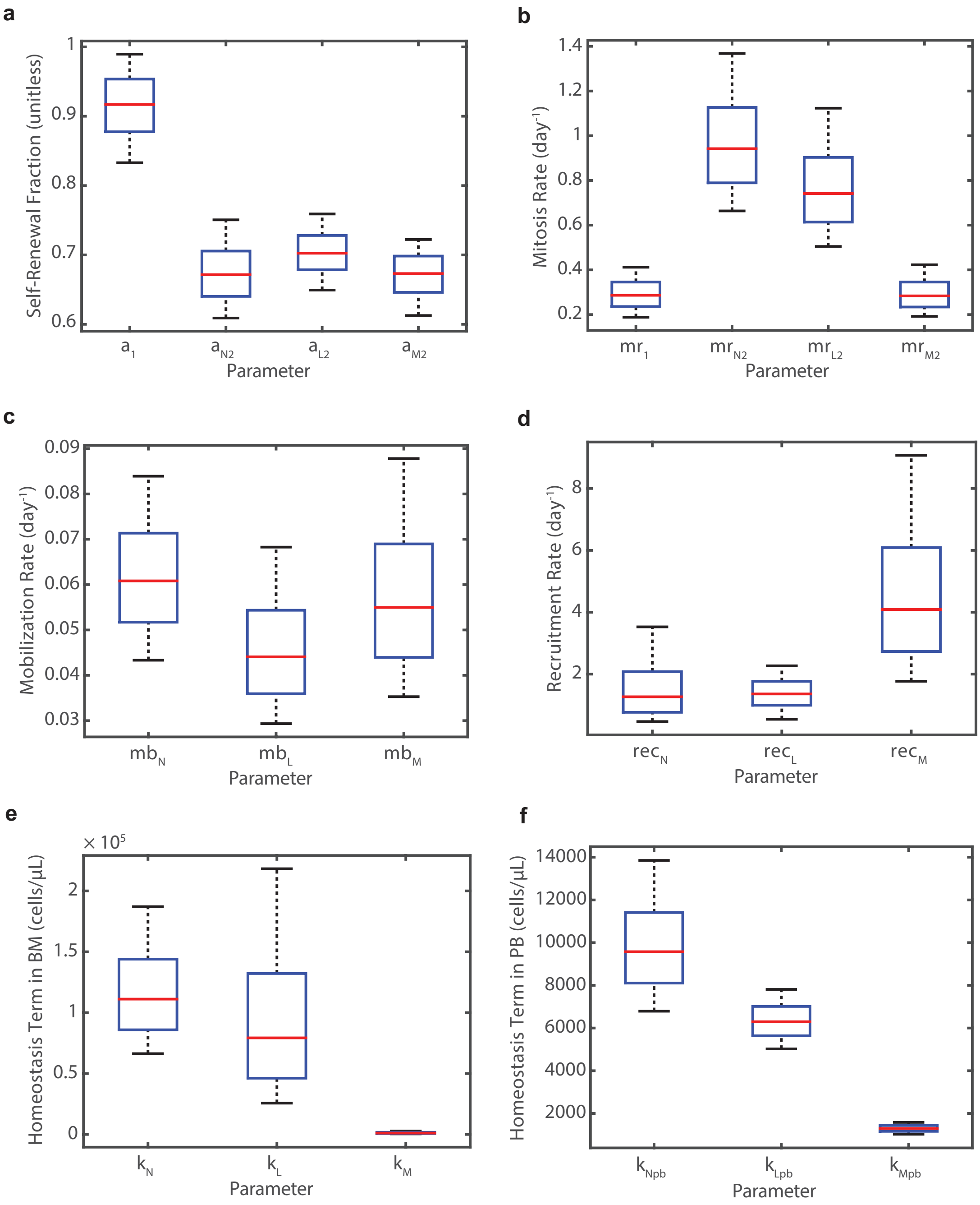

To identify correlations amongst the various lineages, both in parameter space and initial condition (I.C.) space, we computed correlation coefficients for all hematopoieitic non-cancer states and param-

eters in our model of all multi-lineage acceptable simulations (Figure S5). As a reminder, we found initial condition and parameter values simultaneously. We found that strong correlations exist between initial conditions and parameters of analogous lineages, and weak correlations exist between different lineages. However, stem cell parameters are slightly correlated with the initial condition of stem cells and neutrophils in the bone marrow. This is especially true of k_1, the Michaelis-Menten constant that drives stem cells to maintain homeostasis of the whole system.

Figure S5: **Correlation Coefficient Heatmap of Initial Conditions and Parameters.** The row and column show initial conditions (I.C.) and parameters (Param.) in the same order. The initial conditions of the states are in the same order of the first 12 equations in Supplement 2. Namely, the stem cell initial condition is followed by neutrophil (Neut), lymphocyte (Lymp), and monocyte (Mono) initial conditions. Within each category of initial conditions, the progenitor, mature cell in the marrow, the mature cell in the peripheral blood, and the mature cell in the marginal pool (if applicable) are shown. After the initial conditions are compared, the next values are parameters in the order presented in Table S7. White boxes group all initial conditions of the same cell types together. Black boxes group all parameters of the same cell types together. Finally, dark purple boxes group initial conditions and parameters of the same cell type together. The color bar scale for the value of the correlation coefficient is displayed on the right.

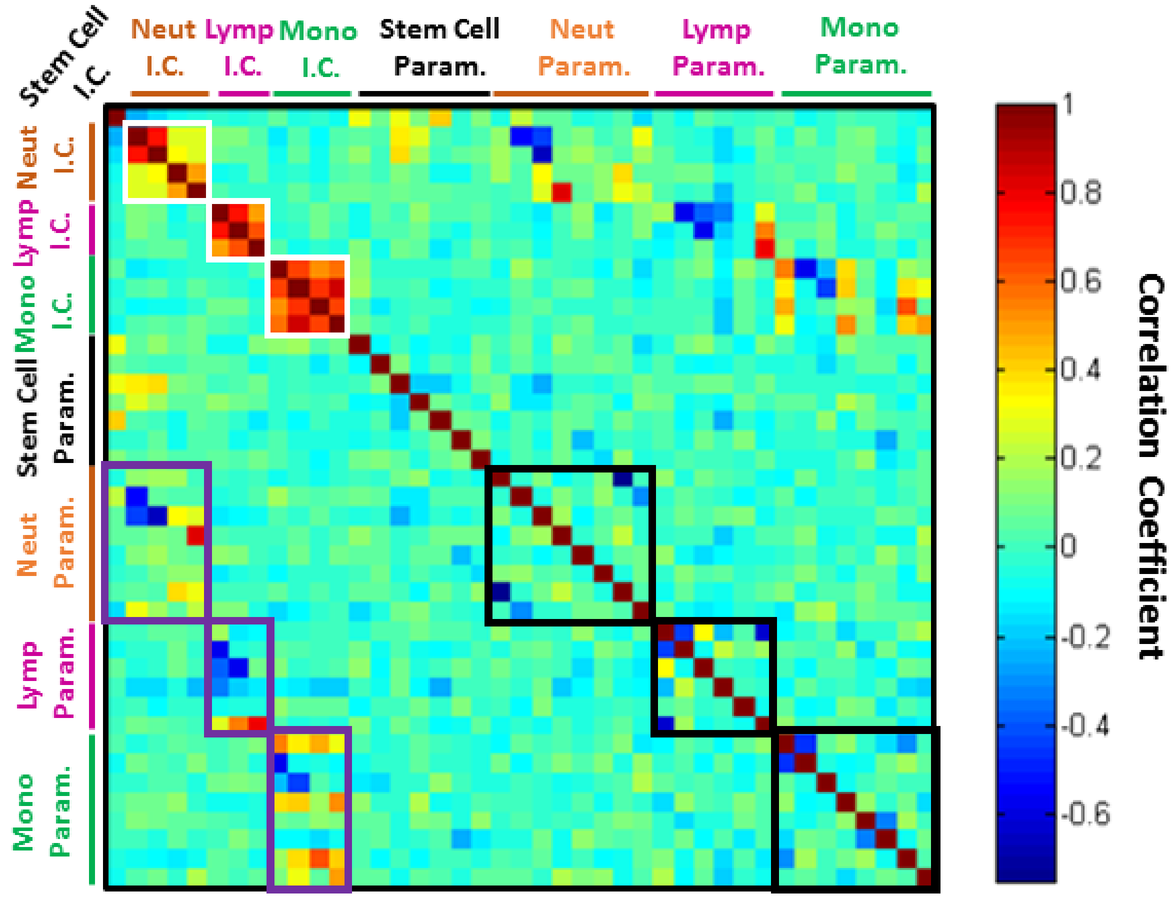

References

[1] A. H. Schmaier and H. M. Lazarus, editors. *Concise Guide to Hematology.* Wiley-Blackwell, West Sussex, UK, 2012.

[2] Anna Marciniak-Czochra and Thomas Stiehl. Mathematical models of hematopoietic reconstitution after stem cell transplantation. *Model Based Parameter Estimation*, 2013.

[3] X. Wang, J. Shook, M. Edinger, N. Warner, and C. Bush-Donovan. Multiparametric immunophenotyping of human hematopoietic stem cells and progenitor cells by flow cytometry. *BD Biosciences*, January 2012.

[4] Hematopoietic stem cells. `http://http://stemcells.nih.gov/info/scireport/pages/chapter5.aspx`, June 2001. Accessed: 2016-04-05.

[5] C. Wayne Smith. Production, distribution, and fate of neutrophils. In K. Kaushansky, M. A. Lichtman, J. T. Prchal, M. M. Levi, O.W. Press, L. J. Burns, and M. Caligiuri, editors, *Williams Hematology*, chapter 61. McGraw-Hill, New York, NY, 2015.

[6] In *Wintrobe's Clinical Hematology*, chapter Appendix A:IV. Lea and Febiger, Philadelphia, PA, 9e edition, 1993.

[7] Tao Le and Vikas Bhushan, editors. *First Aid for the USMLE Step 1 2012*. McGraw-Hill Education, New York, 2012.

[8] Emile van den Akker, Timothy J. Satchwell, Stephanie Pellegrin, Geoff Daniels, and Ashley M. Toye. The majority of the in vitro erythroid expansion potential resides in cd34 cells, outweighing the contribution of cd34+ cells and significantly increasing the erythroblast yield from peripheral blood samples. *Haematologica*, 95, 2010.

[9] K. Takumi, J. Garssen, R. de Jonge, W. de Jong, and A. Havelaar. Release kinetics and cell trafficking in relation to bacterial growth explain the time course of blood neutrophils and monocytes during primary salmonella infection. *International Immunology*, 17:85–93, January 2005.

[10] Georg A. Holländer, Barbara Widmer, and Steven J. Burakoff. loss of normal thymic repertoire selection and persistence of autoreactive t cells in graft vs host disease. *Journal of Immunology*, 152:1609–1617, 1994.

[11] H. Moore and N. K. Li. A mathematical model for chronic myelogenous leukemia (cml) and t cell interaction. *Journal of Theoretical Biology*, 227:513–23, April 2004.

[12] Ralph van Furth, Marina M. C. Diesselhoff-Den Dulk, and Herman Mattie. Quantitative study on the production and kinetics of mononuclear phagocytes during an acute inflammatory reaction. *The Journal of Experimental Medicine*, 138, 1973.

[13] D. M. Whitelaw and H. F. Batho. The disribution of monocytes in the rat. *Cell Tissue Kinetics*, 5:215–225, 1972.

[14] Timo Heidt, Gabriel Courties, Partha Dutta, Hendrik B Sager, Matt Sebas, Yoshiko Iwamoto, Yuan Sun, Nicolas Da Silva, Peter Panizzi, Anja M van der Laan, et al. Differential contribution of monocytes to heart macrophages in steady-state and after myocardial infarctionnovelty and significance. *Circulation research*, 115(2):284–295, 2014.

Supplementary Information 5: Physiological Capabilities of Model

1 Summary of Patient Data

Forty-seven adult patients who underwent peripheral blood stem cell transplants at the IU Simon Cancer Center under the supervision of Dr. Robert P. Nelson, Jr. for the treatment of AML were used to determine patient dynamics in response to chemotherapy. Peripheral blood samples were collected from the patients daily for seven days prior to transplantation, and for thirty days post-transplantation. A complete blood count of the peripheral blood demonstrated neutrophil, lymphocyte, and monocyte concentrations in the peripheral blood. These patients received a non-myeloablative treatment protocol consisting of cyclophosphamide for two days, and fludarabine for the 5 subsequent days. The donor infusion is administered after the chemotherapy regimen is completed.

We do not simulate transplantation within our model. We use the daily blood counts of patients to characterize cell dynamics in the peripheral blood during chemotherapy and qualitative characteristics of patient recovery after chemotherapy. We use transplantation data because more discrete data is available for patients after treatment for a longer time period. The real patients have received transplantation from donors, but we assume that the behaviors of the final cell concentrations would be the same between patients undergoing only chemotherapy and those undergoing transplantation in our simulations. We do not characterize the timed dynamics of patient recovery from transplantation or chemotherapy in the context of the current model.

2 Overshoot

In dynamic acceptability criteria #6, the overshoot of recovery post-chemotherapy in patients was required to be less than 12 times the overshoot value five days post-overshoot. Some simulations displayed overshoot more than we expected, so we wanted to constrain to normal physiological overshoot. As shown in Figure S5, the only one patient demonstrated overshoot of 12 times in the neutrophil lineage. The majority of patients had less than a 6-factor overshoot. Thus, we restricted our criteria to a factor of 12 change for overshoot.

In observing the final multi-lineage simulations (Figure 6 in text, left column, blue lines), the majority of simulations do not overshoot. For those simulations that do overshoot, the overshoot is within a factor of 3 change. This indicates that though a overshoot of 12 times its equilibrium value was used to constrain the parameter space, a smaller overshoot criteria would have been acceptable. To discriminate for each lineage qualitatively in the patient data, only a small number of patients demonstrate neutrophil overshoot though neutrophils have the potential to overshoot the most. Our multi-lineage model analogously shows the smallest amount of overshoot amongst the lineages, and in the uni-lineage model, neutrophils have the potential to overshoot the most (Figure 6b). Additionally, a small percentage of patients demonstrate lymphocyte and/or monocyte overshoot, which we also show in our model (Figure 6c and d).

3 Dampened Oscillations

Simulations that are manually classified as sufficiently dampened versus not dampened are shown in Figure S6. Here, the simulation that does not have a dampened oscillation (Figure S6a) has period of about 60 days, which is longer than physiologically expected for a patient recovering from chemotherapy. On the other hand, a simulation that we manually classifed as acceptable (Figure S6b) has a very high frequency oscillation period after recovering from chemotherapy. After manually classifying 100

Figure S5: **Histogram of Patient Overshoot Distribution.** The overshoot was calculated for the three lineages of data from 47 patients. Neutrophil overshoot is in blue; lymphocyte overshoot is in green; monocyte overshoot is in yellow.

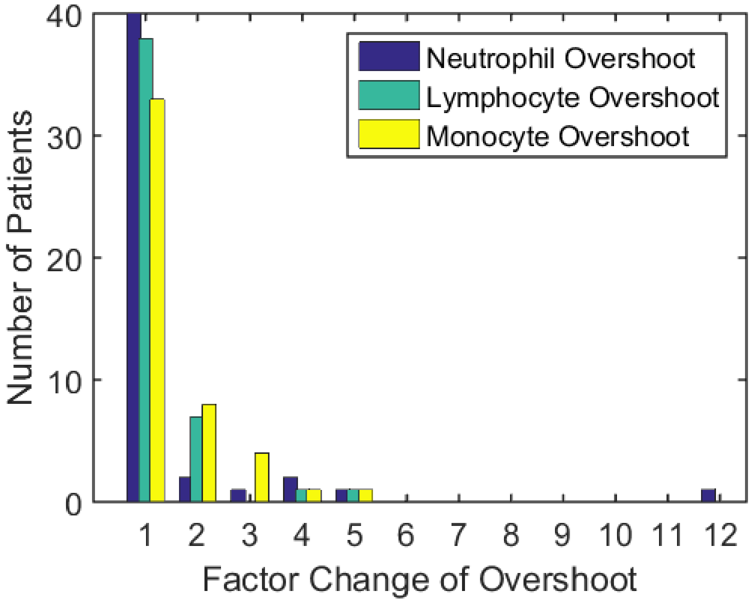

simulations as physiologically acceptable, we found that dynamic criteria #7 uniquely allocated the manually classified simulations correctly.

Figure S6: **Characteristic of unacceptable and acceptable solutions in model.** (a) An unacceptable solution (red) with high amplitude and low frequency oscillation is shown. (b) An acceptable solution (blue) with some oscillation and overshoot during recovery after chemotherapy is shown, but the oscillation meets the dynamical acceptability criteria.

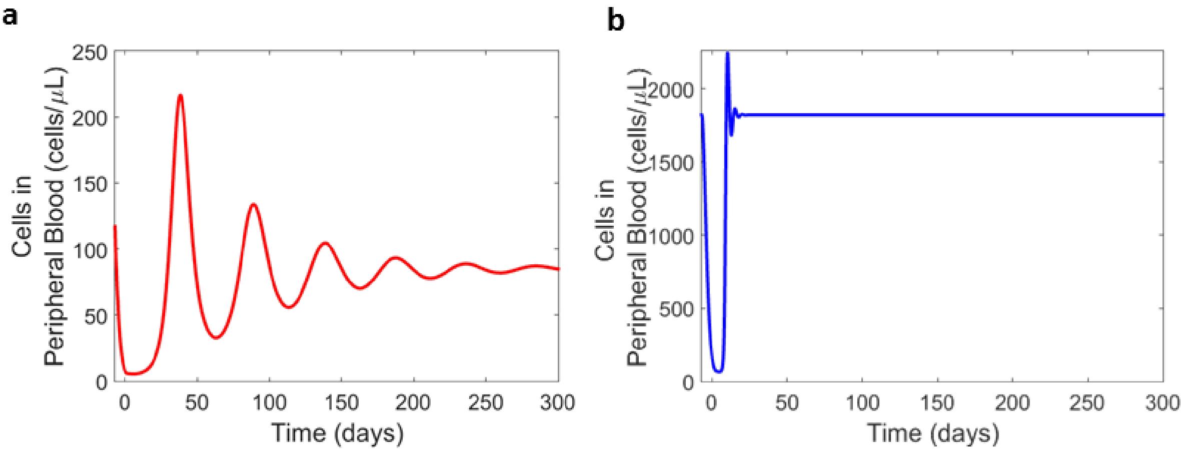

Supplementary Information 6: Sensitivity Analysis

1 Robustness of Multi-Lineage Model

We analyzed the robustness of our model by testing the difference in decoupling the lineages and perturbing one state. We used the 13 centroids of our clustering solutions to change the initial condition of neutrophil progenitors to 10 times its initial value. The results are shown in Figure S7. In the multi-lineage model, this tempers the overshoot of the neutrophil concentrations in the peripheral blood (Figure S7a) but causes oscillations in lymphocyte concentrations in the peripheral blood (Figure S7b). However, in the uni-lineage model, the neutrophils demonstrate much higher overshoots (Figure S7c), and there are no changes in the lymphocyte concentrations (Figure S7d). We do not show monocytes because the changes in their concentrations are minimal with respect to their concentration. This confirms that combining all three lineages provides some robustness to the system.

Figure S7: **Perturbation in Neutrophil Progenitors in Multi-Lineage System Compared to Uni-Lineage System.** The effects of the perturbation of neutrophil progenitors for the 13 most representative patients (demonstrated using different colors analogous to those in Figures 7 and 8 in main text) are shown in (a) neutrophils in the peripheral blood in the multi-lineage system, (b) lymphocytes in the peripheral blood in the multi-lineage system, (c) neutrophils in the peripheral blood in the de-coupled system, and (d) lymphocytes in the peripheral blood in the decoupled system.

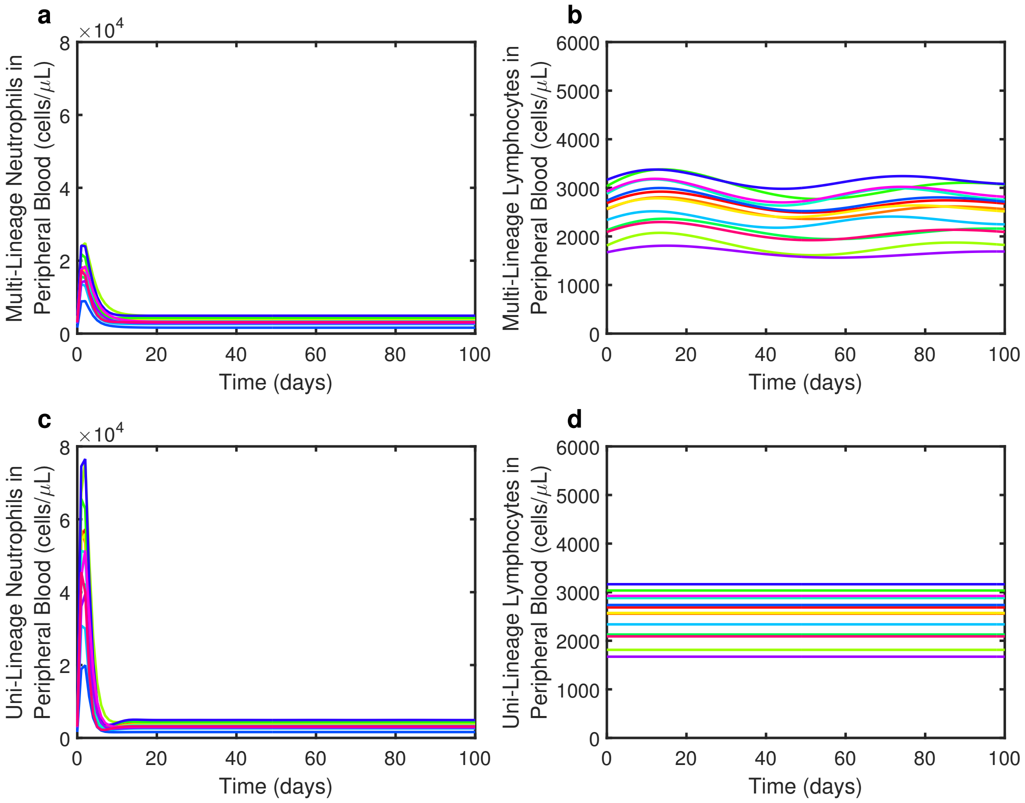

2 Partial Rank Correlation Coefficient (PRCC)

Descriptions and analysis of the Partial Rank Correlation Coefficient (PRCC) global sensitivity analysis are explained in the main text.

Figure S8: **Sensitive Parameters for Stem Cell State.**

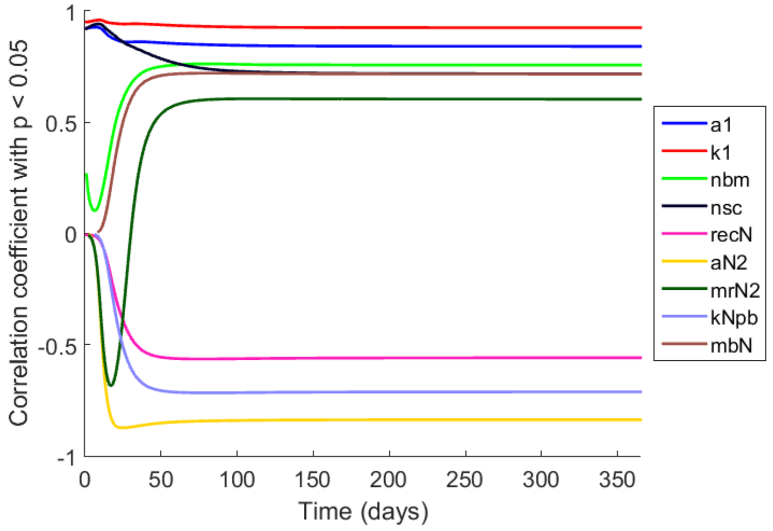

Figure S9: **Sensitive Parameters for Neutrophils in Peripheral Blood State.**

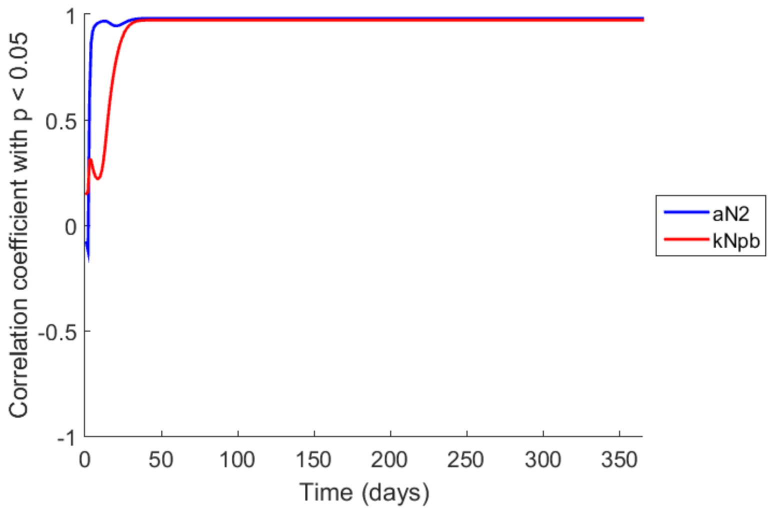

Figure S10: **Sensitive Parameters for Lymphocytes in Peripheral Blood State.**

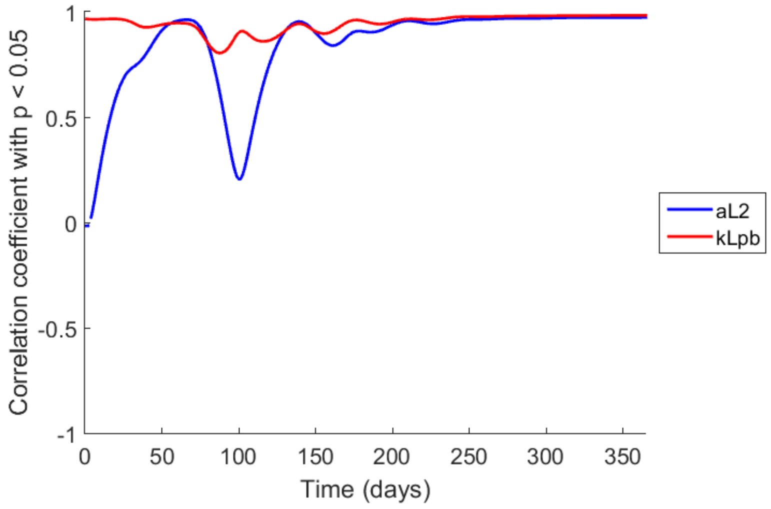

Figure S11: **Sensitive Parameters for Monocytes in Peripheral Blood State.**

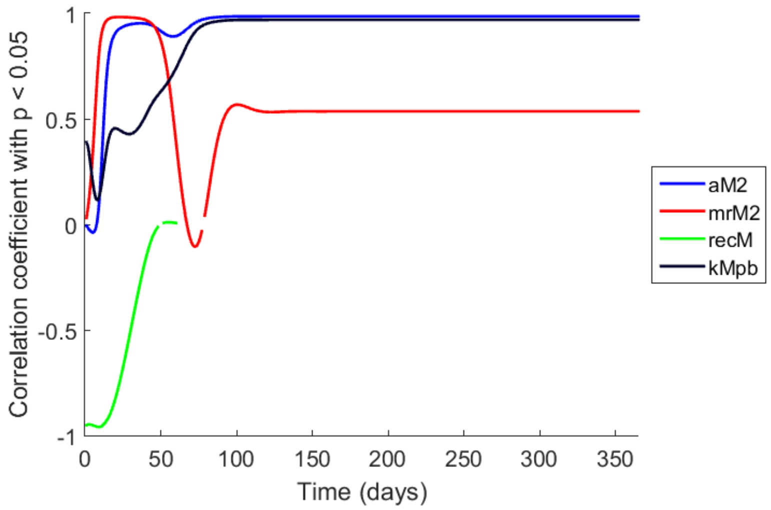

Supplementary Information 7: Determining Cluster Number

K-means clustering requires that the number of clusters be pre-defined before computing the clusters. However, determining the number of clusters to group data is a complex problem. One method is to use the Calinski-Harabasz criteria, or F-statistic, to determine the number of clusters that maximizes the between-cluster variance, but minimizes the within-cluster variance [1]. The Calinski-Harabasz criterion is defined as

$$\text{F-statistic} = \frac{\sum_{i=1}^{k} n_i ||m_i - m||^2}{\sum_{i=1}^{k} \sum_{x \in c_i} ||x - m_i||^2} * \frac{N-k}{k-1} \quad (1)$$

where k is the number of clusters, i is the cluster index, n_i is the number of elements in cluster i, m_i is the centroid of cluster i, m is the overall mean of the data, N is the number of samples in the data, and x is a data point in the ith cluster, represented by c_i.

As the number of clusters increases, the within-cluster variance increases, and in order to ensure that the smallest k is chosen that still produces an acceptable within-cluster variance, a curve can be plotted of the F-statistic against the number of clusters. The optimal cluster number can be found at the elbow of this curve. The elbow of the curve is found by first drawing a line between the two ends of the curve. The point that has the maximum distance between the line and the curve defines k, the optimal cluster number. We wanted to ensure that at least one hundred representatives were in each cluster, so we clustered the data over one cluster to 228 clusters (for the 22,796 representatives; Figure S12). Due to computational time, some of the clusters were not computed. The optimal number of clusters, k, was determined to be 13 (Figure S12).

Figure S12: **Determining Optimal Cluster Number.The F-statistic is maximized as the number of clusters increases (blue curve).** The elbow of the curve determines the optimal number of clusters, which converged to 8 clusters (red +).

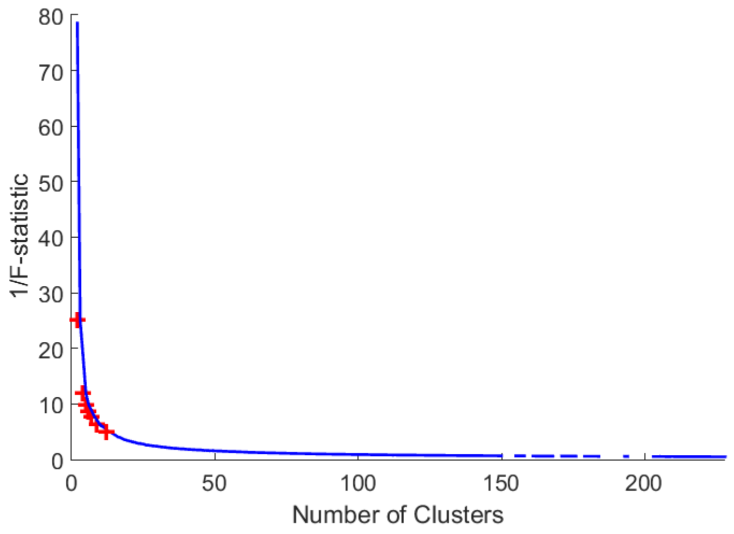

References

[1] T. Calinski and J. Harabasz. A dendrite method for cluster analysis. *Communications in Statistics*, 3:1–27, 1974.

Supplementary Information 8: Neutrophil-Derived AML

Neutrophils and monocytes share a common progenitor. AML can arise when the feedback mechanisms of proliferation and/or differentiation are disrupted in a common myeloid progenitor or a granulocyte-monocyte progenitor [1]. Thus to demonstrate the differences between AML derived from neutrophil progenitors versus those derived from monocyte progenitors, we modeled AML growth from neutrophil progenitors that do not respond to any feedback signals. This is analagous to how we modeled AML derived from monocytes. We find that AML derived from neutrophils grows much more rapidly and is more likely to lead to faster death in patients who have neutrophil-derived AML (Figure S13a-c). This is probably due to the faster mitosis rate of neutrophil progenitors than monocyte progenitors that we determined in Table S7 in Supplement 4. Additionally, due to signaling of homeostastic mechanisms to stem cells and normal progenitors, the cell counts of normal cells remain lower in a neutrophil-derived AML, but the overall white blood cell counts would be higher earlier in these patients' time courses. For one subtype of AML, overall high white blood counts are predictive of survival [2], which is what these results indicate.

However, we confirm that the mitosis rate of the AML is the most correlated parameter to time to death for patients with AML derived from neutrophil progenitors with an overall correlation coefficient of -0.64 (Figure S13d). Additionally, the probability of self-renewal is the second most correlated parameter to survival time of patients with neutrophil-derived AML with an overall correlation coeffeicient of -0.36 (Figure S13d). This corresponds to the same results we found for monocyte-derived AML, and confirms the potential target of treating AML as the probability of self-renewal of progenitor cells.

Figure S13: **Survival in neutrophil-derived leukopoesis.** Each of the thirteen clusters is represented with a distinct color in a-c, corresponding to the same colors as in Figure 7 in the body of the paper. (a) Progenitor cancer stem cells derived from neutrophil progenitors grow over one year in each of the 22,796 simulations. The gray lines represents the cell concentration and time in which patients may die due to overgrowth of cancer cells. (b) The percentage of cells in the peripheral blood due to AML in each of the 22,796 simulations. Patients die prior to reaching 100% peripheral blasts, as indicated by gray lines where simulation continued. In (a) and (b), some cluster simulations are not visible because they are underneath the other simulations. (c) The simulated survival curves of each of the thirteen representative clusters of patients are shown assuming the patient dies when either cancer in the bone marrow or the peripheral blood reaches a concentration greater than 3×10^5 cells/μL. The cumulative survival is shown as a decimal that represents the fraction of patients that are still alive at the time shown on the x-axis.(d) Significant cancer parameter correlation to number of days until death due to bone marrow or peripheral blood density greater than 3×10^5 ($p < 0.05$). Gray boxes represent insignificant correlations, and the correlation values for each of the thirteen clusters are depicted with the color bar.

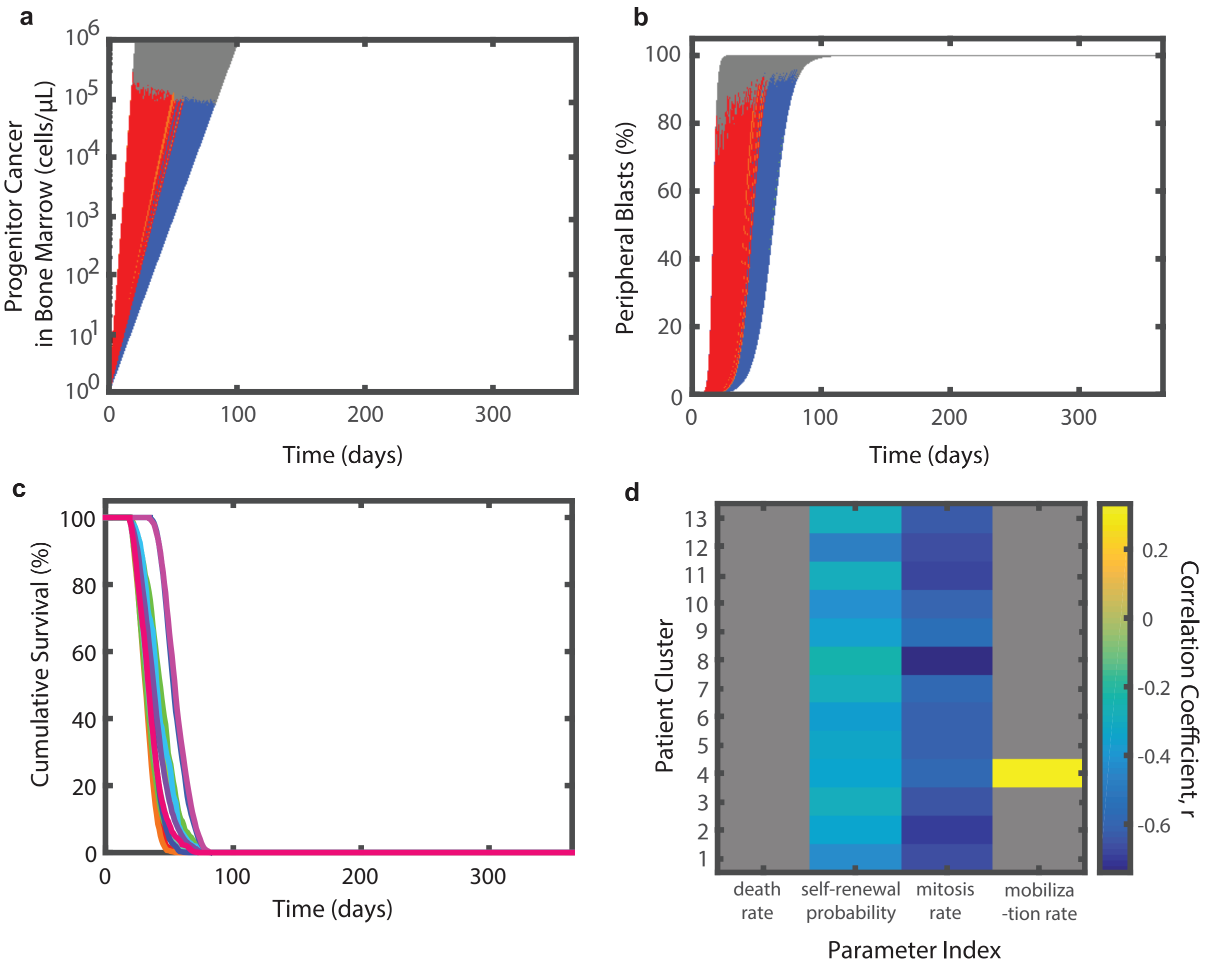

References

[1] Daniel G Tenen. Disruption of differentiation in human cancer: Aml shows the way. *Nature reviews cancer*, 3(2):89–101, 2003.

[2] Stéphanie Nguyen, Thierry Leblanc, Pierre Fenaux, Francis Witz, Didier Blaise, Arnaud Pigneux, Xavier Thomas, Françoise Rigal-Huguet, Bruno Lioure, Anne Auvrignon, et al. A white blood cell index as the main prognostic factor in t (8; 21) acute myeloid leukemia (aml): a survey of 161 cases from the french aml intergroup. *Blood*, 99(10):3517–3523, 2002.
